# Exercise preserves β-cell function in type 2 diabetes by reshaping intra-islet macrophage–β-cell crosstalk

**DOI:** 10.1093/lifemeta/loag014

**Published:** 2026-05-26

**Authors:** Miqi Yang, Yanping Zhou, Qing-Qian Wu, Wenjing Zhang, Rui Zhang, Zhuoying Yang, Ting Yu, Ruo-Ran Wang, Hongxing Fu, Qi Fu, Di Chen, Zhuo-Xian Meng, Zhe Yu Zhang

**Affiliations:** Department of Pathology and Pathophysiology and Department of Hepatobiliary and Pancreatic Surgery of the Second Affiliated Hospital, Zhejiang University School of Medicine, Hangzhou, Zhejiang 310009, China; Department of Geriatrics, Hangzhou First People’s Hospital, Hangzhou, Zhejiang 310006, China; Department of Pathology and Pathophysiology and Department of Hepatobiliary and Pancreatic Surgery of the Second Affiliated Hospital, Zhejiang University School of Medicine, Hangzhou, Zhejiang 310009, China; Department of Geriatrics, Hangzhou First People’s Hospital, Hangzhou, Zhejiang 310006, China; Department of Pathology and Pathophysiology and Department of Hepatobiliary and Pancreatic Surgery of the Second Affiliated Hospital, Zhejiang University School of Medicine, Hangzhou, Zhejiang 310009, China; Department of Geriatrics, Hangzhou First People’s Hospital, Hangzhou, Zhejiang 310006, China; Center for Reproductive Medicine of The Second Affiliated Hospital, Center for Regeneration and Cell Therapy of Zhejiang University-University of Edinburgh Institute (ZJU-UoE Institute), Zhejiang University School of Medicine, Zhejiang University, Hangzhou, Zhejiang 310003, China; Department of Pathology and Pathophysiology and Department of Hepatobiliary and Pancreatic Surgery of the Second Affiliated Hospital, Zhejiang University School of Medicine, Hangzhou, Zhejiang 310009, China; Department of Geriatrics, Hangzhou First People’s Hospital, Hangzhou, Zhejiang 310006, China; Department of Pathology and Pathophysiology and Department of Hepatobiliary and Pancreatic Surgery of the Second Affiliated Hospital, Zhejiang University School of Medicine, Hangzhou, Zhejiang 310009, China; Department of Geriatrics, Hangzhou First People’s Hospital, Hangzhou, Zhejiang 310006, China; Department of Pathology and Pathophysiology and Department of Hepatobiliary and Pancreatic Surgery of the Second Affiliated Hospital, Zhejiang University School of Medicine, Hangzhou, Zhejiang 310009, China; Department of Geriatrics, Hangzhou First People’s Hospital, Hangzhou, Zhejiang 310006, China; Department of Pathology and Pathophysiology and Department of Hepatobiliary and Pancreatic Surgery of the Second Affiliated Hospital, Zhejiang University School of Medicine, Hangzhou, Zhejiang 310009, China; Department of Geriatrics, Hangzhou First People’s Hospital, Hangzhou, Zhejiang 310006, China; Department of Hepatobiliary and Pancreatic Surgery of the Second Affiliated Hospital, Zhejiang University School of Medicine, Hangzhou, Zhejiang 310009, China; Department of Endocrinology & Metabolism, The First Affiliated Hospital with Nanjing Medical University, Nanjing, Jiangsu 210029, China; Center for Reproductive Medicine of The Second Affiliated Hospital, Center for Regeneration and Cell Therapy of Zhejiang University-University of Edinburgh Institute (ZJU-UoE Institute), Zhejiang University School of Medicine, Zhejiang University, Hangzhou, Zhejiang 310003, China; Edinburgh Medical School: Biomedical Sciences, College of Medicine and Veterinary Medicine, The University of Edinburgh, Edinburgh EH8 9YL, United Kingdom; State Key Laboratory of Biobased Transportation Fuel Technology, Haining, Zhejiang 314400, China; Dr. Li Dak Sum & Yip Yio Chin Center for Stem Cell and Regenerative Medicine, Zhejiang University, Hangzhou, Zhejiang 310003, China; Zhejiang Key Laboratory of Medical Imaging Artificial Intelligence, Haining, Zhejiang 314400, China; Biomedical and Health Translational Research Center of Zhejiang Province, Haining, Zhejiang 314400, China; Department of Pathology and Pathophysiology and Department of Hepatobiliary and Pancreatic Surgery of the Second Affiliated Hospital, Zhejiang University School of Medicine, Hangzhou, Zhejiang 310009, China; Department of Geriatrics, Hangzhou First People’s Hospital, Hangzhou, Zhejiang 310006, China; Affiliated Huzhou Hospital, Zhejiang University School of Medicine, Huzhou, Zhejiang 313000, China; Department of Pathology and Pathophysiology and Department of Hepatobiliary and Pancreatic Surgery of the Second Affiliated Hospital, Zhejiang University School of Medicine, Hangzhou, Zhejiang 310009, China; Department of Geriatrics, Hangzhou First People’s Hospital, Hangzhou, Zhejiang 310006, China

**Keywords:** exercise intervention, diabetes, β-cell, metabolic disorders, SPARC

## Abstract

Type 2 diabetes (T2D) is characterized by pancreatic islet β-cell dysfunction and systemic insulin resistance, with meta-inflammation playing a critical role in disease progression. As the major type of immune cell population in islets, both resident and recruited macrophages are important regulators of the islet immune microenvironment under physiological and T2D conditions. Exercise is an effective strategy for treating T2D, yet its impacts on islet inflammation and β-cell dysfunction remain elusive. Here, we established a mouse model of exercise intervention in obesity-associated T2D by combining high-fat diet (HFD) feeding with treadmill running. Notably, exercise markedly improves glucose tolerance and insulin sensitivity, accompanied by substantial mitigation of HFD-induced β-cell dysfunction, islet hypertrophy, and alterations in β-cell subpopulations. Exercise also reduces intra-islet infiltration of CD45^+^ immune cells and dampens pro-inflammatory gene expression, indicating robust attenuation of islet inflammation. Using untargeted plasma proteomics, we identified the secreted protein acidic and rich in cysteine (SPARC) as a circulating factor, whose suppression is associated with exercise-linked islet protection under HFD conditions. Mechanistically, our data support a model in which SPARC contributes to β-cell dysfunction, at least in part, through macrophage inflammasome-related signaling. Further analysis of a human cohort demonstrates that circulating SPARC protein levels are markedly elevated in patients with T2D, exhibiting a significant negative correlation with parameters indicative of insulin sensitivity and β-cell function, and a positive correlation with insulin resistance. Together, this work provides a systemic characterization of the effects of exercise intervention on islet homeostasis and β-cell function, and highlights SPARC as a candidate immuno-metabolic node for T2D intervention.

## Introduction

Type 2 diabetes (T2D) is an escalating global health crisis, cha­racterized by pancreatic islet β-cell dysfunction and systemic insulin resistance [1]. The progressive decline of β-cell function in T2D is closely linked to the dysregulation of the islet immune microenvironment [1], in which both islet-resident and recruited immune cells play pivotal yet mechanistically elusive roles. Far from being isolated clusters of merely endocrine cells, pancrea­tic islets constitute a complex cellular niche that hosts diverse immune components, including macrophages, which are essential for maintaining tissue homeostasis under physiological conditions [2–4]. However, under chronic metabolic stress, such as obesity and high-fat diet (HFD) feeding, the islet landscape and immune profile undergo profound remodeling [5–7]. This is cha­racterized by the local expansion and pro-inflammatory activation of intra-islet macrophages [8], which, together with systemic insults such as hyperglycemia and elevated free fatty acids, trigger oxidative and endoplasmic reticulum (ER) stress in β-cells [9–13]. Although these pathophysiological processes ultimately culminate in β-cell failure and apoptosis [14–18], the precise molecular drivers that govern the interaction between immune remodeling and β-cell dysfunction remain to be fully elucidated. As such, unraveling the molecular basis of islet immune remodeling and adaptation is essential for developing targeted therapeutic strategies to preserve or restore β-cell function and halt T2D progression.

Beyond its well-established role in systemic insulin sensitization [19], physical exercise has emerged as a potent modulator of β-cell plasticity, likely acting through complex inter-organ crosstalk [20, 21]. Skeletal muscle and other peripheral tissues release endocrine signals, such as myokines [21–23], adipokines [24–26], and hepatokines [27–29], which orchestrate adaptive responses in distant organs, including pancreatic islets. These signals may enhance β-cell survival and secretory capacity by mitigating the glucolipotoxic and pro-inflammatory milieu of T2D [26, 30, 31]. Recent clinical studies have demonstrated that lifestyle interventions, such as physical exercise, are effective in both preventing and treating T2D [32, 33]. Moreover, previous studies have highlighted β-cell plasticity, especially the recovery of the first-phase of insulin secretion, as a critical determinant of T2D progression and remission [34–36]. However, although exercise is a well-recognized intervention for preserving β-cell function [37], whether its protective effects involve remodeling of the islet immune microenvironment remains largely unknown. As such, it is critical to elucidate the mechanisms by which exercise intervention prevents or delays T2D progression. Given the technical challenges of directly assessing islet homeostasis in humans, the establishment of a robust murine model is essential for investigating how exercise reshapes the immuno-metabolic niche of the islets to preserve or restore β-cell function during the development of T2D.

Previously, we established a murine model that recapitulates the dynamic β-cell adaptation during T2D progression by feeding C57BL/6J wild-type (WT) mice with an HFD for different durations. Using this model, we provided a systemic multi-omics characterization of islet homeostasis and β-cell function during T2D progression [9]. Building on these findings, the present study comprehensively characterizes the protective effects of treadmill exercise on islet homeostasis and β-cell function during T2D development. By integrating physiological assessments with single-cell transcriptomics, we demonstrated that exercise intervention induced profound β-cell remodeling, effectively reversing maladaptive subpopulation shifts and restoring functional β-cell identity. Furthermore, we showed that exercise reshaped the intra-islet immune landscape by attenuating pro-inflammatory CD45^+^ immune cell infiltration and resolving the chronic inflammatory microenvironment triggered by metabolic stress. Through systemic proteomic profiling, we identified the secreted protein acidic and rich in cysteine (SPARC) as a candidate exercise-regulated circulating factor whose suppression is associated with the islet-protective phenotypes. A previous study reported that adipocyte-derived SPARC promotes NOD-like receptor family pyrin domain-containing 3 (NLRP3) inflammasome at the priming step in macrophages through c-Jun N-terminal kinase (JNK)/p38 mitogen-activated protein kinase (p38) (JNK/p38) signaling [38]. In line with this, we further investigated whether SPARC contributes to the subsequent inflammasome activation step in macrophage under metabolic stress. Our findings support a model in which exercise-associated downregulation of circulating SPARC helps attenuate a deleterious macrophage-to-β-cell signaling axis, thereby preserving β-cell secretory function and maintaining normal β-cell subpopulation composition. Moreover, human cohort studies confirmed the clinical relevance of circulating SPARC levels in patients with T2D, revealing a significant negative correlation with indices of insulin sensitivity and β-cell function, and a positive correlation with insulin resistance para­meters. Together, our work establishes a multi-dimensional framework linking physical exercise to islet protection and highlights the SPARC-associated macrophage–β-cell axis as a pathway of potential translational interest.

## Results

### Exercise intervention improves metabolic health in T2D mice

**Figure 1:**
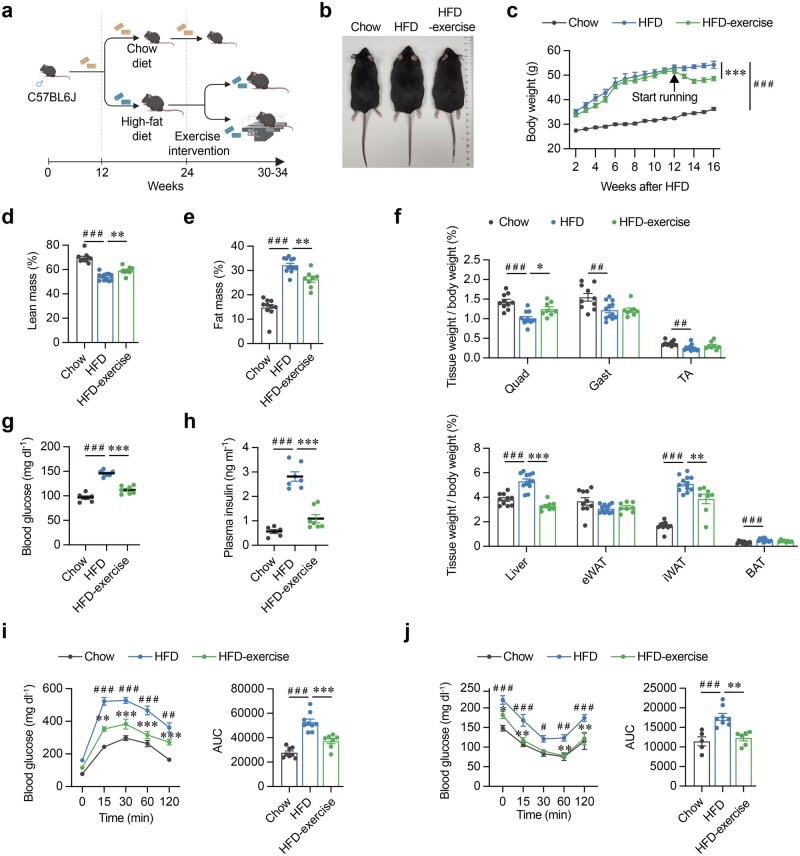
Exercise intervention ameliorates HFD-induced metabolic abnormalities. (a) Schematic diagram of the experimental setup. (b) Representative images of mice in Chow, HFD, and HFD-exercise groups. (c) Body weight of mice with treatments indicated in (a and b). Data represents mean ± SEM (*n *= 6–12 mice per group). ^###^*P *< 0.001, HFD vs. Chow; ***P < 0.001, HFD-exercise vs. HFD; two-way ANOVA with Tukey’s multiple comparison test. (d) Lean mass percentage of mice in the indicated groups. Data represent mean ± SEM (*n *= 8–12 mice per group). ^###^*P *< 0.001, HFD vs. Chow; **P < 0.01, HFD-exercise vs. HFD; one-way ANOVA with Tukey’s multiple comparison test. (e) Fat mass percentage of mice in the indicated groups. Data represent mean ± SEM (*n *= 8–12 mice per group). ^###^*P *< 0.001, HFD vs. Chow; **P < 0.01, HFD-exercise vs. HFD; one-way ANOVA with Tukey’s multiple comparison test. (f) Quad, gast, TA, liver, eWAT, iWAT, and BAT weight of mice from Chow, HFD, and HFD-exercise groups. Data represents mean ± SEM (*n *= 8–12 mice per group). ^###^*P *< 0.001 and ^##^*P *< 0.01, HFD vs. Chow; ***P < 0.001, **P < 0.01, *P < 0.05, HFD-exercise vs. HFD; one-way ANOVA with Tukey’s multiple comparison test. (g) Overnight fasting blood glucose levels at 8 weeks after exercise intervention. Data represent mean ± SEM (*n *= 6–7 mice per group). ^###^*P *< 0.001, HFD vs. Chow; ***P < 0.001, HFD-exercise vs. HFD; one-way ANOVA with Tukey’s multiple comparison test. (h) Overnight fasting blood insulin levels at 8 weeks after exercise intervention. Data represent mean ± SEM (*n *= 6–7 mice per group). ^###^*P *< 0.001, HFD vs. Chow; ***P < 0.001, HFD-exercise vs. HFD; one-way ANOVA with Tukey’s multiple comparison test. (i) Intraperitoneal GTT at 4 weeks after exercise intervention (left). Data represent mean ± SEM (*n *= 7–9 mice per group). ^###^*P *< 0.001 and ^##^*P *< 0.01, HFD vs. Chow; ***P < 0.001 and **P < 0.01, HFD-exercise vs. HFD; two-way ANOVA with Tukey’s multiple comparison test. AUC quantification of GTT results (right). ^###^*P *< 0.001, HFD vs. Chow; ***P < 0.001, HFD-exercise vs. HFD; one-way ANOVA with Tukey’s multiple comparison test. (j) Intraperitoneal ITT at 5 weeks after exercise intervention (left). Data represent mean ± SEM (*n *= 5–8 mice per group). ^###^*P *< 0.001, ^##^*P *< 0.01, ^#^*P *< 0.05, HFD vs. Chow; **P < 0.01 and *P < 0.05, HFD-exercise vs. HFD; two-way ANOVA with Tukey’s multiple comparison test. AUC quantification of ITT results (right). ^###^*P *< 0.001, HFD vs. Chow; **P < 0.01, HFD-exercise vs. HFD; one-way ANOVA with Tukey’s multiple comparison test. Data in (c–j) are representative of at least two independent experiments. AUC, area under the curve; BAT, brown adipose tissue; eWAT, epididymal white adipose tissue; Gast, gastrocnemius muscle; iWAT, inguinal white adipose tissue; Quad, quadriceps; TA, tibialis anterior muscle.

In this study, we utilized an HFD-induced murine model to investigate the effects of exercise intervention on β-cell function during T2D progression. Three-month-old male C57BL/6J WT mice were fed either a standard chow diet (Chow) or an HFD for 3 months, a period that encompasses the transition from β-cell compensation to decompensation accompanied by extensive transcriptomic remodeling [9]. HFD-fed mice were then randomized into a sedentary group (HFD) or an exercise intervention group (HFD-exercise), with the latter subjected to treadmill running for additional 6–10 weeks while maintained on HFD (Fig. 1a). This regi­men was designed to mimic the exercise intervention during the prediabetic to early T2D stages in the previously published clinical study [39]. HFD feeding induced marked body weight gain relative to Chow-fed controls, whereas exercise substantially attenuated this weight gain, resulting in intermediate body weights in the HFD-exercise group (Fig. 1b and c). Consistent with these findings, body composition analyses demonstrated that exercise partially corrected HFD-induced alterations in metabolic tissue mass, including increased liver and inguinal white adipose tissue (iWAT) mass and reduced quadriceps mass (Fig. 1d–f).

We next assessed systemic glucose homeostasis. HFD feeding induced fasting hyperglycemia and hyperinsulinemia, both of which were markedly ameliorated by exercise intervention (Fig. 1g and h). Glucose tolerance tests (GTTs) revealed pronounced glucose intolerance in HFD mice, as indicated by an increased area under the curve (AUC), whereas exercise substantially improved glucose tolerance in HFD-exercise mice (Fig. 1i). Similarly, insulin tolerance tests (ITTs) showed that exercise significantly alleviated HFD-induced insulin resistance (Fig. 1j). Collectively, these data demonstrate that exercise intervention during chronic overnutrition attenuates HFD-induced obesity, partially normalizes metabolic tissue composition, and restores systemic glucose homeostasis.

### Exercise preserves islet morphology and β-cell function in T2D mice

**Figure 2:**
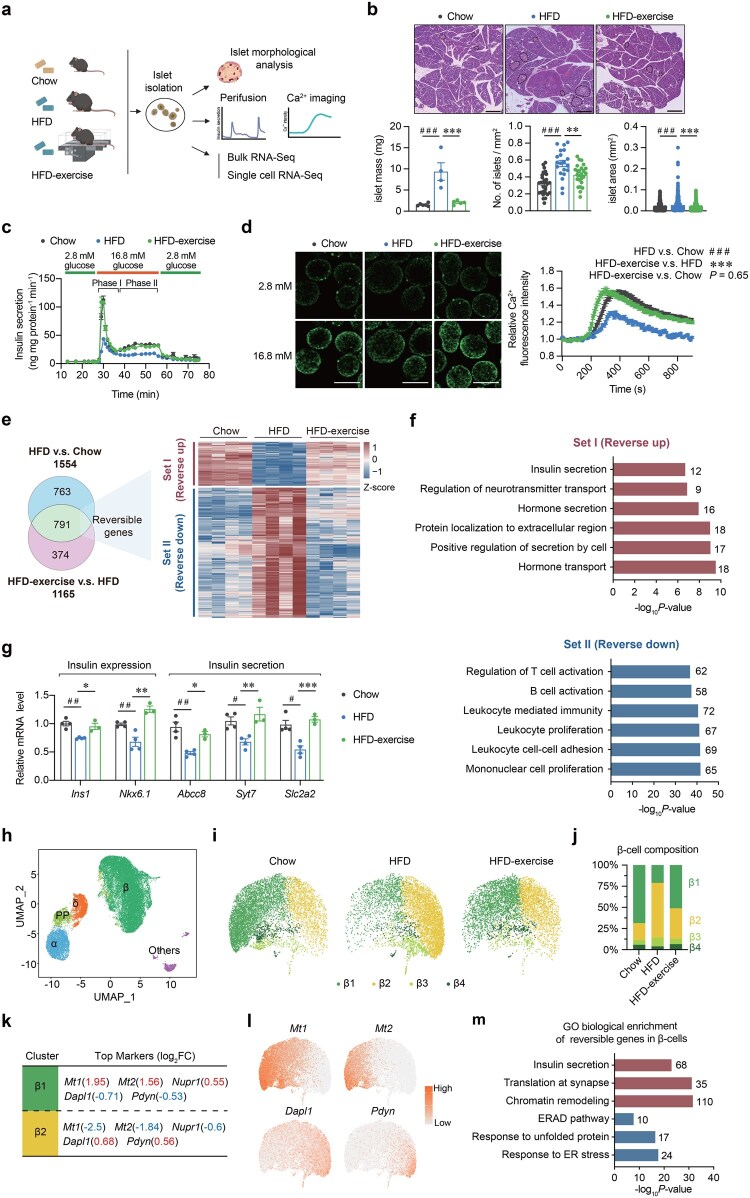
Exercise preserves islet homeostasis and β-cell function in diabetic mice. (a) Schematic diagram of the experimental setup. (b) Representative images of H&E-stained pancreatic sections from Chow, HFD, and HFD-exercise groups (up). Scale bar, 500 µm. Quantitative analysis of islet mass, islet number per pancreas section, and islet area per pancreas section from Chow, HFD, and HFD-exercise mouse islets (down). Each sample consisted of tissues pooled from 4–6 mice. Data represent mean *±* SEM (islet number per pancreas section: *n = *20–30 pancreatic sections from each group; islet area per pancreas section: *n = *377–472 islets from each group). ^###^*P *< 0.001, HFD vs. Chow; ***P < 0.001, **P < 0.01, HFD-exercise vs. HFD; one-way ANOVA with Tukey’s multiple comparison test. (c) Perifusion analysis of dynamic glucose-stimulated insulin secretion in islets from Chow, HFD, and HFD-exercise mice. Each islet sample was pooled from at least three animals. Data represent mean *±* SD (*n *= 3 technical replicates). (d) Representative IF images of high glucose-stimulated (16.8 mmol/L) Ca^2+^ flux in mouse islets isolated from Chow (*n *= 86 islets), HFD (*n *= 68 islets), and HFD-exercise (*n *= 67 islets) groups (left). Each islet sample was pooled from at least three animals. Scale bar, 200 μm. Dynamic Ca^2+^ fluorescence intensity in response to high-glucose treatment (right). Data represent mean ± SEM. ^###^*P *< 0.001, HFD vs. Chow; ***P < 0.001, HFD-exercise vs. HFD; two-way ANOVA with Tukey’s multiple comparison test. (e) Venn plot showing the overlap of differential genes (left). Relative content heatmap for overlapped, reversible genes of mice in the indicated groups (right) (*n *= 4 mice per group). (f) GO analysis of reversible genes indicated in (e). (g) Relative mRNA levels of islet function-associated genes in islets from Chow, HFD, and HFD-exercise mice. Data represent mean *±* SEM (*n = *3–4 biological replicates per group). ^##^*P *< 0.01 and ^#^*P *< 0.05, HFD vs. Chow; ***P < 0.001, **P < 0.01, and *P < 0.05, HFD-exercise vs. HFD; one-way ANOVA with Tukey’s multiple comparison test. (h) scRNA-seq and UMAP visualization of transcriptome similarity-based cell clustering of islet single cells from indicated groups. Islet sample for each treatment was pooled from at least three animals. In total, 11 646, 8747, and 7235 cells in Chow, HFD, and HFD-exercise groups, respectively, were used in the clustering. (i) UMAP visualization of β-cell subclusters in indicated groups. Cells apart from the major β-cell subcluster on the UMAP plot are not shown. (j) Composition analysis of β-cell subclusters in indicated groups. (k) Top markers of indicated β-cell subclusters. Upregulated genes were marked in red, while downregulated genes were marked in blue. log_2_(FC) indicates the average log_2_-transferred fold change of indicated gene expression in given subcluster comparing to the expression level in other β-cells. (l) Expression heatmap of indicated genes in all β-cells. (m) GO analysis of reversible genes (with *P *< 0.05 by Wilcoxon Rank Sum test) in β-cells. Most significant and nonredundant biological processes with respective gene numbers and *P* values are shown. Reverse up-regulated and reverse down-regulated terms are indicated in red and blue, respectively. Data in (b–g) are representative of at least two independent experiments.

To investigate the impact of exercise intervention on pancreatic islet morphology and function, as well as the underlying mechanisms, we isolated islets from Chow, HFD, and HFD-exercise mice for comprehensive assessment (Fig. 2a). Hematoxylin and eosin (H&E) staining of pancreatic sections revealed that HFD induced robust islet hyperplasia and hypertrophy, resulting in an approxi­mately 4- to 5-fold increase in total islet mass compared with Chow controls, accompanied by significant increases in both islet size and number per unit area (Fig. 2b; Supplementary Fig. S1a). In contrast, exercise intervention markedly reversed these morphological alterations, effectively restoring islet size and number toward normal levels (Fig. 2b). Immunofluorescence (IF) analysis of pancreatic sections from mice after 10 weeks of exercise intervention further confirmed these changes (Supplementary Fig. S1b). Furthermore, quantitative analysis of the proportion of insulin-expressing β-cells in pancreatic sections revealed a significant reduction in the HFD group compared with the Chow group, which was effectively reversed by exercise intervention (Supplementary Fig. S1b). These findings suggest that, under prolonged HFD exposure, despite compensatory islet hyperplasia and hypertrophy, the proportion of functionally competent β-cells is substantially reduced, providing a potential explanation for the marked decline in islet function during the decompensated phase. Importantly, these maladaptive structural changes can be reversed by exercise intervention.

To delineate the dynamic insulin secretory profiles of islets from the three experimental groups, we employed a self-assembled islet perifusion system [9] to monitor real-time insulin secretion during both first and second phases in response to glucose stimulation. Compared with Chow islets, HFD islets exhibited significant impairment in both phases of insulin secretion. Exercise intervention largely restored these biphasic secretory dynamics to near-normal levels (Fig. 2c; Supplementary Fig. S1c). Glucose stimulated insulin secretion (GSIS) in pancreatic β-cells is a classic example of “metabolism-electrical activity-secretion” coupling, whereby glucose metabolism closes ATP-sensitive potassium (K_ATP_) channels, induces plasma membrane depolarization, opens voltage-dependent Ca^2+^ channels, and triggers Ca^2+^ influx-dependent insulin granule exocytosis [40]. To investigate whether treadmill exercise preserves β-cell insulin secretion via the K_ATP_ channel-dependent pathway, we isolated primary islets from the three mouse groups and loaded them with the Ca^2+^-sensitive fluorescent dye Fluo-4 AM in the presence of Pluronic F-127. Upon glucose stimulation, Chow islets showed a mean peak Ca^2+^ response of 1.555, whereas the HFD islets displayed a significantly blunted response, with a mean peak of only 1.311. Notably, islets from the HFD-exercise group exhibited substantial recovery, reaching a mean peak Ca^2+^ signal of 1.574 (Fig. 2d). Together, these findings suggest that early exercise intervention effectively preserves and restores β-cell insulin secretory capacity, at least in part through enhancing K_ATP_ channel-dependent calcium signaling.

Next, we performed bulk RNA-seq analysis of isolated islets and identified 791 genes that were reversibly regulated by both HFD feeding and exercise intervention (Fig. 2e). Gene Ontology (GO) enrichment analysis of these reversible genes showed predominant enrichment in pathways related to insulin secretion and inflammatory response (Fig. 2f). Independent reverse transcription-quantitative polymerase chain reaction (RT-qPCR) validation further confirmed that exercise intervention effectively reversed the HFD-induced downregulation of genes involved in insulin expression and secretion (Fig. 2g).

We next investigated the impact of exercise intervention on pancreatic β-cell heterogeneity using microwell-based single-cell RNA sequencing (scRNA-seq). Islet cells were clustered into five major populations and annotated according to the expression of characteristic endocrine hormone genes as α-cells, β-cells, δ-cells, PP-cells, and a non-endocrine cluster composed primarily of immune and endothelial cells. Uniform Manifold Approximation and Projection (UMAP) visualization confirmed that the analyzed islet cells were predominantly endocrine, with relatively low proportions of immune and endothelial cells (Fig. 2h). To precisely delineate β-cell subpopulations for downstream analysis, we visualized the expression of the β-cell marker genes *Ins1* and *Ins2* in cells from the exercise intervention model. Both *Ins1* and *Ins2* were highly and specifically expressed within the annotated β-cell cluster (Supplementary Fig. S1d). Given that GSIS function prima­rily depends on β-cells [41], we performed subclustering analysis of the identified β-cell population, which revealed four distinct subpopulations, designated as β1–β4. HFD feeding induced a profound shift in β-cell composition, markedly decreasing the proportion of the β1 subpopulation from 68.6% in Chow group to 21% in HFD group, while increasing the β2 subpopulation from 20% in Chow group to 64.9% in HFD group. These changes were largely reversed by exercise intervention (Fig. 2i and j). The β1 and β2 subpopulations represented the predominant subsets, collectively accounting for approximately 90% of all β-cells.

To elucidate the mechanistic basis underlying the functional differences between β1 and β2 subpopulations, we performed comparative transcriptomic analysis across the four β-cell subclusters. This analysis identified several genes with specific expression patterns that define the β1 and β2 identities. The β1 subpopulation was characterized by predominant expression of metallothionein 1 (*Mt1*), metallothionein 2 (*Mt2*), and nuclear protein 1 (*Nupr1*), whereas the β2 subpopulation was characterized by elevated expression of death-associated protein-like 1 (*Dapl1*) and prodynorphin (*Pdyn*) (Fig. 2k). Previous studies have established that Mt1 and Nupr1 exert protective effects in pancreatic β-cells against oxidative stress, inflammatory insults, and other stress-induced damage [42–44]. Consistent with these roles, our analysis indicated that the β1 subcluster exhibited molecular features associated with greater stress resistance and better insulin secretion capacity compared to β2 cells (Fig. 2l). Moreover, elevated *Dapl1* and *Pdyn* expression in the β2 subcluster suggest heightened cellular stress and activation of an opioid peptide-related negative feedback program, which may contri­bute to impaired insulin secretion under metabolic stress [45, 46] and provide a mechanistic explanation for their impaired GSIS relative to their β1 cells (Fig. 2l).

To further investigate the effects of exercise intervention on the β-cell transcriptome, we performed differential gene expression analysis across all β-cells, defining significantly altered genes using a cutoff of *P* value < 0.05. Compared with the Chow group, the HFD group exhibited upregulation of 415 genes, which were significantly enriched in biological processes such as the ER stress response. In parallel, 3596 genes were downregulated in HFD β-cells, with predominant enrichment in pathways involved in the regulation of insulin secretion (Fig. 2m). Moreover, among the 4011 genes dysregulated by HFD, exercise intervention successfully reversed the expression of 2194 genes, which we defined as “reversible genes.” Among these genes, exercise restored the HFD-induced downregulation of insulin secretion-related genes, including *Kcnj11*, *Slc2a2*, *Vamp2*, and *Ucn3*, as well as the antioxidant gene *Mt1*. Conversely, several ER stress-related genes, including *Creld2*, *Preb*, *Hspa5*, *Pdia4*, and *Txnip*, exhibited the opposite expression pattern (Supplementary Fig. S1e). Collectively, these findings indicate that exercise intervention reshapes β-cell subpopulation composition and transcriptomic states, shifting β-cells toward a protected and functionally competent phenotype that helps maintain islet homeostasis.

### Exercise intervention mitigates islet inflammation in T2D mice

**Figure 3:**
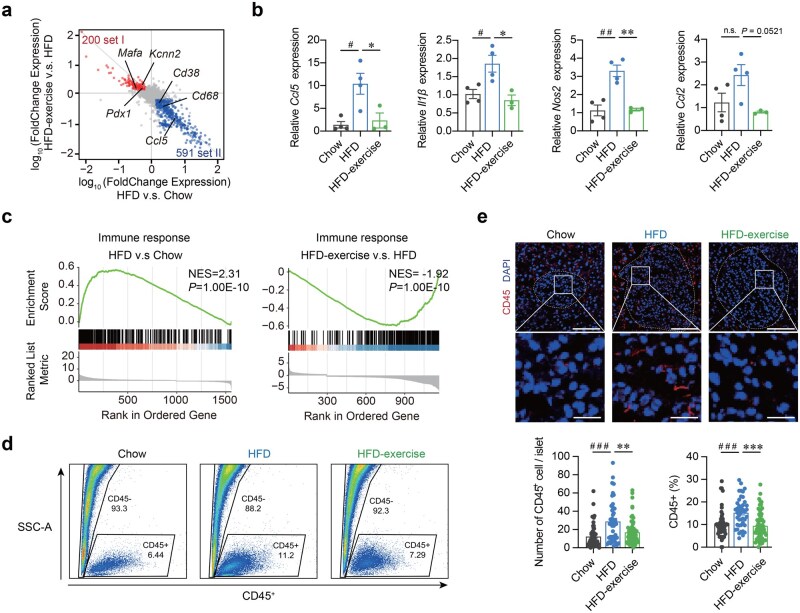
Exercise ameliorates islet inflammation in HFD-fed mice. (a) Scatter plot of differentially expressed genes from RNA-seq analysis of mouse islet across Chow, HFD, and HFD-exercise groups. log_10_-transferred fold change expression levels of all highly expressed genes (fragments per kilobase of exon model per million mapped fragments > 1) in indicated comparisons are shown in scatter plot. Reversible genes with |log_2_(FC)| > 0.5, *P *< 0.05 were additionally marked in red (upregulated) and blue (downregulated) (*n *= 4 mice per group). (b) Relative mRNA levels of pro-inflammation-associated genes in islets from Chow, HFD, and HFD-exercise mice. Data represent mean *±* SEM (*n = *3–4 biological replicates per group). ^##^*P *< 0.01 and ^###^*P *< 0.05, HFD vs. Chow; **P < 0.01 and *P < 0.05, HFD-exercise vs. HFD; n.s., no significance; one-way ANOVA with Tukey’s multiple comparison test. (c) GSEA of differentially expressed immune response-related genes in islets from Chow, HFD, and HFD-exercise mice. (d) Representative flow cytometry plots and quantification of CD45^+^ cells from islets of the indicated groups. Islets were pooled from at least three animals per group and digested into single cells for analysis. (e) Representative IF images of CD45 (red) in islets from Chow, HFD, and HFD-exercise groups (up). Scale bar, 100 µm (overview) and 20 µm (inset). Quantitative analysis of the number and the proportion of CD45^+^ cells from Chow, HFD, and HFD-exercise mouse islets (down). Each sample consisted of tissues pooled from 4–6 mice. Data represent mean *±* SEM (dots represent 43–62 islets from each group). ^###^*P *< 0.001, HFD vs. Chow; ***P < 0.001 and **P < 0.01, HFD-exercise vs. HFD; one-way ANOVA with Tukey’s multiple comparison test. All panels report data in at least two independent experiments. NES, normalized enrichment score.

To elucidate the impact of exercise intervention on the islet immune microenvironment, we performed transcriptomic profiling of islets isolated from Chow, HFD, and HFD-exercise mice. Compared with Chow islets, HFD islets exhibited a distinct molecular signature, with 989 genes upregulated and 565 genes downregulated (Supplementary Fig. S2a). GO enrichment analysis revealed that HFD-upregulated genes were predominantly associated with immune cell activation and proliferation, whereas downregulated genes were enriched in pathways governing insulin transport and secretory function (Supplementary Fig. S2b). Notably, treadmill exercise largely normalized the HFD-induced transcriptional shifts. Comparison between the HFD-exercise and HFD islets identified 294 upregulated and 871 downregulated genes (Supplementary Fig. S2c), with functional enrichment patterns largely parallel to, but opposite from, those observed in the HFD versus Chow comparison (Supplementary Fig. S2d). Specifically, we identified 791 “reversible” genes whose expression was altered by HFD but significantly restored by exercise (Fig. 2e), among which 591 HFD-induced genes were subsequently downregulated following exercise intervention (Fig. 3a). Consistent with the GO enrichment analysis (Fig. 2f), RT-qPCR validation confirmed that exercise intervention markedly attenuated the HFD-induced transcriptional surge of key pro-inflammatory marker genes (Fig. 3b). Furthermore, Gene Set Enrichment Analysis (GSEA) demonstrated systemic upregulation of immune response pathways in HFD islets, which was robustly reversed by exercise (Fig. 3c). These findings suggest that exercise induces a comprehensive transcriptional reprogramming in islets, effectively counteracting HFD-driven inflammation while preserving metabolic and secretory homeostasis.

Given the transcriptomic evidence of altered immune signaling, we next quantified immune cell infiltration into the islets. Flow cytometry analysis of dissociated primary islets using the pan-leukocyte marker CD45 revealed that HFD feeding nearly doubled the proportion of islet-resident immune cells, increasing from 6.44% in Chow islets to 11.2% in HFD islets. Remarkably, exercise intervention almost entirely abolished this accumulation, restoring immune cell proportions to near-baseline levels (Fig. 3d).

To further validate these findings *in situ*, we performed IF staining for CD45 on pancreatic sections (Fig. 3e). Qualitative assessment showed a marked increase in CD45^+^ cells both within and surrounding the islets of HFD-fed mice. Quantitative analysis confirmed a 132% increase in the number of CD45^+^ cells per islet and a 52% increase in the ratio of immune cells to total islet cells in the HFD group compared to Chow controls. In alignment with our flow cytometry data, exercise intervention effectively reversed these increases (Fig. 3e). Collectively, these results demonstrate that treadmill exercise intervention prevents HFD-induced immune cell accumulation within the pancreatic islet microenvironment.

### Identification of SPARC as an exercise-regulated factor associated with islet function

**Figure 4:**
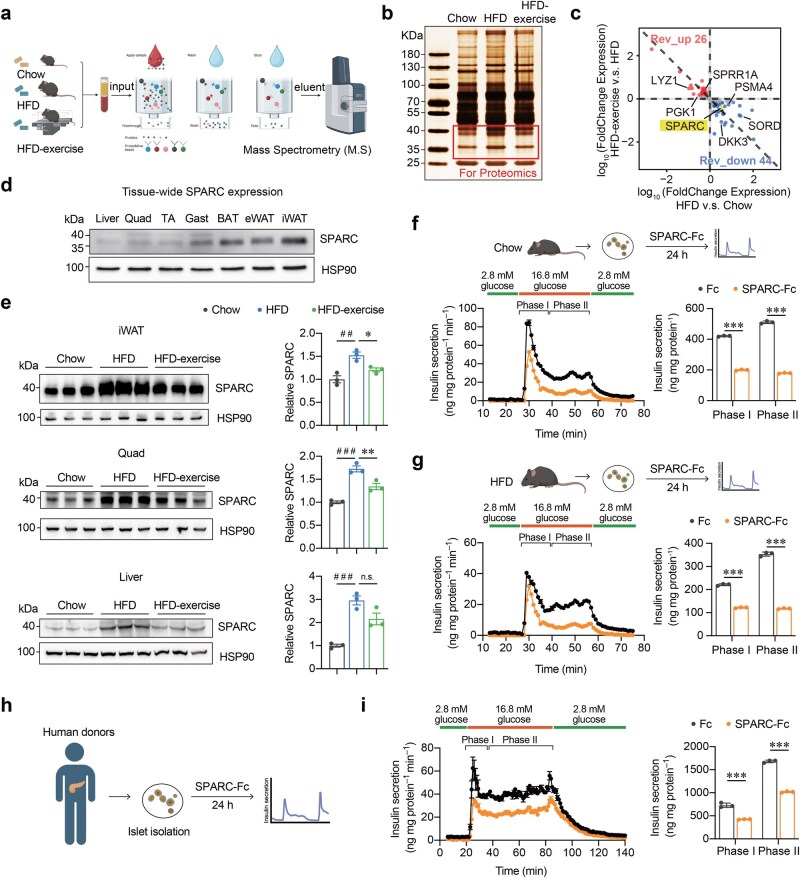
The exercise-regulated circulating factor SPARC impairs islet function. (a) Schematic workflow of the plasma proteomic analysis from mice subjected to a 10-week treadmill exercise intervention. (b) Representative image of sliver-stained plasma proteins following the procedure outlined in (a). The gel bands encased in the red box were excised for subsequent mass spectrometric analysis. Each plasma sample was pooled from at least six mice. (c) Volcano plots of proteins with reversible expression in plasma. Significant changes (|log_2_(FC)| > 0.5) are highlighted in red (upregulated), blue (downregulated), and gray (insignificance). (d) Immunoblots of SPARC from total protein lysates of liver, Quad, TA, Gast, BAT, eWAT, and iWAT from WT mice. (e) Immunoblots and quantitative analyses of total protein lysates from iWAT, Quad, and liver of Chow, HFD, and HFD-exercise mice. Data represent mean ± SEM (*n *= 3 mice per group); ^###^*P *< 0.001 and ^##^*P *< 0.01, HFD vs. Chow; **P < 0.01 and *P < 0.05, HFD-exercise vs. HFD; n.s., no significance; one-way ANOVA with Tukey’s multiple comparison test. (f and g) Perifusion analyses of dynamic glucose-stimulated phase I and phase II insulin secretion in islets collected from Chow (f) and HFD (g) mice after 24-h *ex* *vivo* SPARC treatment. Each islet sample was pooled from at least three animals. Data represent mean *±* SD (*n *= 3 technical replicates). ***P < 0.001; two-way ANOVA with Tukey’s multiple comparison test. (h) Schematic representation for experimental procedures for (i). (i) Perifusion analyses of dynamic glucose-stimulated phase I and phase II insulin secretion of human islets collected after 24-h *ex* *vivo* SPARC treatment. Each sample was pooled with at least 100 islets. Data represent mean *±* SD (*n *= 3 technical replicates). ***P < 0.001; two-way ANOVA with Tukey’s multiple comparison test. Data in (b–g) and (i) are representative of at least two independent experiments. BAT, brown adipose tissue; eWAT, epididymal white adipose tissue; Gast, gastrocnemius; iWAT, inguinal white adipose tissue; Quad, quadriceps; TA, tibialis anterior.

To investigate systemic mediators of exercise-induced islet protection, we sought to identify circulating factors involved in inter-tissue communication. Plasma from Chow, HFD, and HFD-exercise mice was pretreated using bead-based combinatorial peptide ligand library technology to deplete high-abundance proteins and enrich the low-abundance proteins (Fig. 4a). Subsequent silver staining and mass spectrometry analysis of bands corresponding to proteins with exercise-reversible expression identified 164 proteins modulated by exercise intervention (Fig. 4b). Among these, 70 proteins showed significant differential expression in the HFD-exercise group relative to the HFD group, including 26 upregulated and 44 downregulated proteins (Fig. 4c). Within this exercise-responsive proteome, we prioritized SPARC for further investigation (Supplementary Fig. S3a), given its established role in promoting adipose tissue inflammation and its potential to influence systemic immune homeostasis [38, 47]. To determine the potential sources of circulating SPARC, we cha­racterized its expression profile across key metabolic organs. Both transcriptional and protein-level analyses revealed that SPARC was predominantly expressed in the adipose tissue and skeletal muscle, with lower but detectable levels in the liver and pancreas (Fig. 4d; Supplementary Fig. S3b). Importantly, SPARC expression across these metabolic tissues was highly sensitive to nutritional and physical stimuli. HFD feeding significantly induced SPARC expression at both the mRNA and protein levels across all exa­mined tissues, whereas exercise intervention robustly attenuated this induction (Fig. 4e; Supplementary Fig. S3c). These results suggest that SPARC functions as a systemic circulating factor whose levels integrate dietary and exercise-related metabolic cues.

Having identified SPARC as an exercise-modulated circulating factor, we next examined its functional impact on islets. Recombinant human SPARC fused to an Fc tag (SPARC-Fc) was produced in a mammalian 293F eukaryotic expression system, and its purity and identity were confirmed by Coomassie Brilliant Blue staining and immunoblotting (Supplementary Fig. S3d). To investigate the effect of SPARC on insulin secretion, primary islets isolated from both Chow- and HFD-fed mice were treated *ex vivo* with purified SPARC-Fc (or Fc control) protein for 24 h. Perifusion assays revealed that SPARC treatment significantly blunted GSIS capacity, an effect observed in both healthy Chow islets and HFD-stressed islets (Fig. 4f and g). To evaluate the translational relevance of these findings, we extended our studies to primary human islets. Primary islets from healthy human donors were cultured and treated *ex vivo* with SPARC-Fc and Fc control for 24 h (Fig. 4h). Consistent with the findings in murine model, SPARC-Fc treatment significantly impaired GSIS function in human islets compared with Fc controls (Fig. 4i). Collectively, these results demonstrate that SPARC exerts a detrimental effect on β-cell insulin secretion function across species. As such, exercise-mediated reduction in SPARC levels may represent a potential mechanism contributing to the preservation of β-cell function during T2D progression.

### SPARC impairs β-cell function in a macrophage-dependent manner

**Figure 5:**
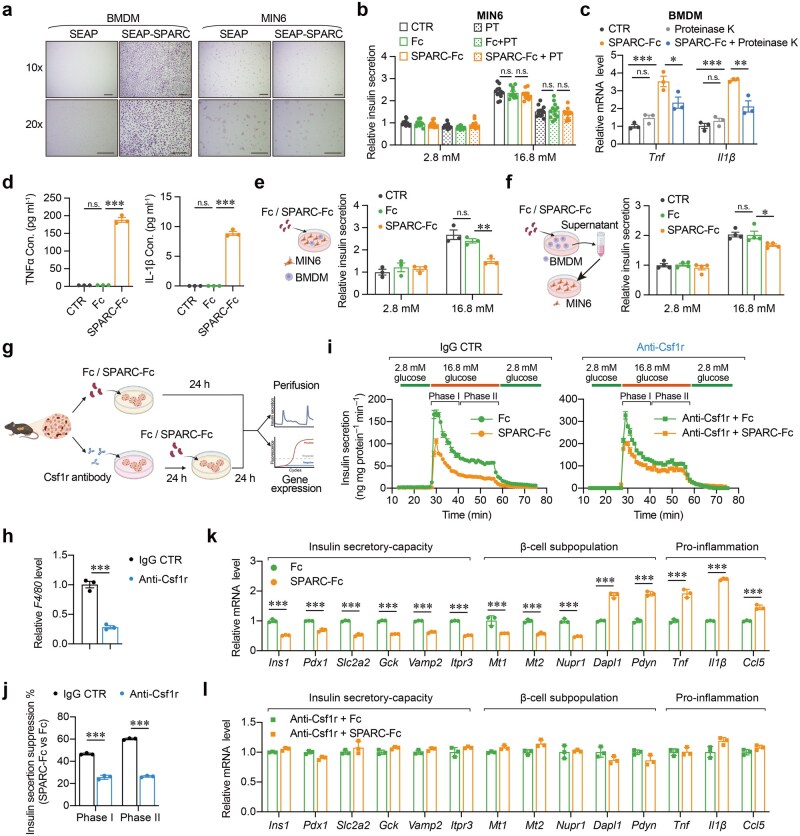
SPARC induces β-cell dysfunction through its action on macrophages. (a) Binding assay on BMDMs and MIN6 cells using SEAP or SEAP-SPARC fusion proteins. Scale bar, 200 μm. (b) GSIS of MIN6 cells cultured with SPARC-Fc or Fc protein, with or without PT treatment. Data represent mean ± SEM (*n *= 12 biological replicates per group). n.s., no significance; two-way ANOVA with Tukey’s multiple comparison test. (c) Relative mRNA levels of inflammatory cytokines in BMDMs treated with SPARC-Fc with or without proteinase K. Data represent mean ± SEM (*n = *3 biological replicates per group). ***P < 0.001; **P < 0.01; *P < 0.05; n.s., no significance; one-way ANOVA with Tukey’s multiple comparison test. (d) TNF-α and IL-1β levels in supernatants from BMDMs stimulated with SPARC-Fc or Fc proteins. Data represent mean *±* SEM (*n = *3 biological replicates per group). ***P < 0.001; n.s., no significance; one-way ANOVA with Tukey’s multiple comparison test. (e) GSIS of MIN6 cells in BMDMs + MIN6 cell co-culture system treated with SPARC-Fc or Fc protein. Data represent mean *±* SEM (*n = *3 biological replicates per group). **P < 0.01; n.s., no significance; two-way ANOVA with Tukey’s multiple comparison test. (f) GSIS of MIN6 cells treated with indicated BMDM supernatants. Data represent mean *±* SEM (*n = *4 biological replicates per group). *P < 0.05; n.s., no significance; two-way ANOVA with Tukey’s multiple comparison test. (g) Schematic representation for experimental procedures for (h–l). (h) Relative *F4/80* gene expression in islets treated with anti-Csf1r antibody and their corresponding controls. Data represent mean ± SEM (*n *= 3 biological triplicates per group). ***P < 0.001; two-tailed unpaired Student’s *t*-test. (i) Perifusion analyses of dynamic glucose-stimulated phase I and phase II insulin secretion of islets treated with SPARC-Fc or Fc proteins (left). Perifusion analyses of dynamic glucose-stimulated phase I and phase II insulin secretion of anti-Csf1r antibody-mediated macrophage depleted islets treated with SPARC-Fc or Fc proteins (right). Data represent mean ± SD (*n *= 3 technical replicates per group). (j) Percentage of the decline in AUC of biphasic insulin release in anti-Csf1r antibody-mediated macrophage depleted islets and their corresponding controls with exogenous SPARC. Data represent mean ± SD (*n *= 3 technical replicates per group). ***P < 0.001; two-tailed unpaired Student’s *t*-test. (k) Relative insulin secretory capacity, β-cell subpopulation, and pro-inflammatory gene expression in islets treated with SPARC-Fc or Fc proteins. Data represent mean ± SEM (*n *= 3 biological triplicates per group). ***P < 0.001; two-tailed unpaired Student’s *t*-test. (l) Relative insulin secretory capacity, β-cell subpopulation, and pro-inflammatory gene expression in macrophage-depleted islets treated with SPARC-Fc or Fc protein. Data represent mean ± SEM (*n *= 3 biological triplicates per group); two-tailed unpaired Student’s *t*-test. Data in (a–f) and (h–l) are representative of at least two independent experiments. SEAP, secreted alkaline phosphatase.

To elucidate the mechanism by which circulating SPARC impairs islet function, we first sought to identify its primary cellular target within the islet niche. Using a secreted alkaline phosphatase (SEAP)-SPARC fusion protein in a cell-binding assay, we observed robust binding activity in bone marrow-derived macrophages (BMDMs) but negligible binding to MIN6 cells, a murine β-cell line [48] (Fig. 5a). Consistent with these findings, exogenous SPARC treatment across a wide concentration range failed to directly alter GSIS in MIN6 cells, either under basal conditions (Supplementary Fig. S3e) or upon challenge with a mixture of palmitic acid and tumor necrosis factor-α (TNF-α) (PT)-induced lipotoxic and inflammatory stress (Fig. 5b). These data demonstrate that SPARC did not exert a direct regulatory effect on β-cell function. We next investigated the impact of SPARC on macrophages. SPARC stimulation induced a dose-dependent upregulation of key pro-inflammatory cytokine genes, including *Tnf* and *Il1β*, in BMDMs (Supplementary Fig. S3e). This response was sensitive to proteolytic degradation, as it was substantially abolished by proteinase K treatment [49] (Fig. 5c). To rule out potential confounding effects of the Fc tag on pro-inflammatory gene expression, we included an Fc-only treatment group. Fc treatment did not alter pro-inflammatory gene expression in BMDMs relative to the untreated control, whereas SPARC-Fc treatment significantly induced these genes compared with the Fc control group (Supplementary Fig. S3h). Furthermore, SPARC significantly enhanced the secretion of TNF-α and interleukin-1β (IL-1β) under basal conditions (Fig. 5d), suggesting that SPARC acts as a potent pro-inflammatory activator of macrophages.

To determine whether SPARC impairs β-cell function through a macrophage-dependent paracrine mechanism, we employed a BMDM and MIN6 cell co-culture system designed to model macrophage–β-cell communication with the intra-islet microenvironment. In this setting, SPARC treatment significantly impaired the GSIS function in MIN6 cells (Fig. 5e). To further confirm that this was driven by soluble factors secreted by SPARC-activated macrophages, we treated MIN6 cells with supernatant from SPARC-activated BMDMs and observed markedly impaired insulin secretion (Fig. 5f). These findings establish that SPARC exerts its deleterious effects on β-cells indirectly by activating macrophages and stimulating the release of pro-inflammatory mediators.

To validate this macrophage-dependent mechanism in a phy­s­iologically relevant context, we performed *ex vivo* studies using primary murine islets. Building on our co-culture findings, we employed an anti-Csf1r antibody to deplete islet-resident macrophages [50, 51] prior to SPARC treatment (Fig. 5g and h). In intact islets, recombinant SPARC markedly suppressed both phase I and phase II insulin secretion during dynamic perifusion assays (Fig. 5i; Supplementary Fig. S3f). Remarkably, depletion of islet-resident macrophages almost completely mitigated these deleterious effects, preserving the biphasic insulin secretion despite the presence of SPARC (Fig. 5i and j; Supplementary Fig. S3g). Transcriptional profiling further elucidated the molecular basis of this rescue. SPARC-induced downregulation of genes critical for insulin secretion function was substantially restored in macrophage-depleted islets (Fig. 5k and l). Notably, SPARC exposure triggered a maladaptive shift in β-cell identity, characterized by the suppression of β1 subcluster maturation markers (*Mt1*, *Mt2*, and *Nupr1*) and reciprocal induction of dysfunctional β2 subcluster genes (*Dapl1* and *Pdyn*). This identity shift was largely prevented by macrophage depletion (Fig. 5l). Furthermore, the SPARC-induced upregulation of pro-inflammatory cytokine genes was significantly attenuated in the absence of macrophage (Fig. 5k and l). Collectively, these results provide strong evidence that islet-resident macrophages are essential cellular mediators of SPARC-induced β-cell dysfunction, driving both transcriptomic remodeling and secretory failure. These findings reinforce the critical role of SPARC-associated macrophage–β-cell axis in metabolic deterioration.

### SPARC induces β-cell dysfunction in T2D mice by activating the NLRP3 inflammasome in macrophages

**Figure 6:**
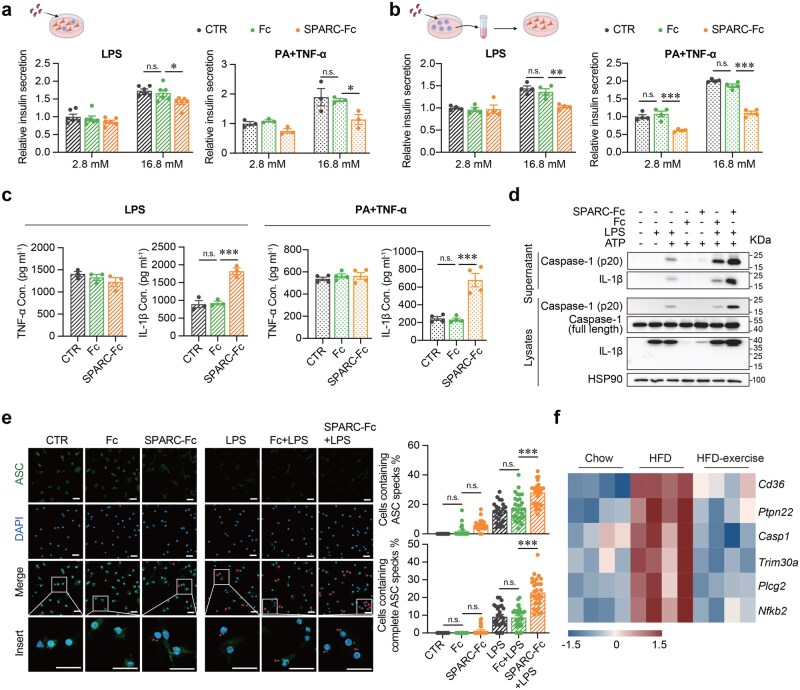
SPARC links metabolic stress to β-cell dysfunction through macrophage NLRP3 inflammasome activation. (a) GSIS of MIN6 cells in BMDMs + MIN6 cell co-culture system treated with SPARC-Fc or Fc protein in LPS or PT condition. Data represent mean *±* SEM (*n = *3–6 biological replicates per group). *P < 0.05; n.s., no significance; two-way ANOVA with Tukey’s multiple comparison test. (b) GSIS of MIN6 cells treated with indicated BMDM supernatants in LPS or PT condition. Data represent mean *±* SEM (*n = *4 biological replicates per group); ***P < 0.001; **P < 0.01; n.s., no significance; two-way ANOVA with Tukey’s multiple comparison test. (c) TNF-α and IL-1β levels in supernatants from BMDMs pretreated with SPARC-Fc proteins followed by stimulation with LPS or PT. Data represent mean *±* SEM (*n = *3–4 biological replicates per group); ***P < 0.001; n.s., no significance; one-way ANOVA with Tukey’s multiple comparison test. (d) Immunoblot analysis of inflammasome activation after pretreatment of SPARC-Fc or Fc protein for 24 h following ATP (1.1 mg/mL) treatment with or without LPS (1 μg/mL) measured by caspase-1 and IL-1β in cell lysate (lower) and supernatant (upper). (e) Representative IF images and quantification of ASC specks (green) in BMDMs pretreated with SPARC-Fc or Fc protein for 24 h, with or without LPS priming (4 h), followed by nigericin (1.5 h) (left). Scale bar, 20 µm. Quantification of the percentage of ASC-speck-positive cells per view (right). Data represent mean ± SEM (*n* = 3 biological replicates per group, each dot represents one field of view). ***P < 0.001; n.s., no significance; one-way ANOVA with Tukey’s multiple comparison test. (f) Relative expression heatmap for the overlap of reversibly expressed, NLRP3 inflammasome-associated genes in islets from mice of the indicated groups (*n* = 4 mice per group). Scale bar shows Z-score. All panels represent data in at least two independent experiments.

To evaluate whether SPARC retains its detrimental effects under multifactorial metabolic stress, we employed a co-culture system mimicking T2D-assocaited stress conditions, incorporating PT-induced lipotoxic and inflammatory stress and lipopolysaccharide (LPS)-induced endotoxemia [52]. Under these metabolic stress conditions, SPARC significantly exacerbated β-cell dysfunction in a macrophage-dependent manner (Figs. 5b and 6a). Consistent with our previous observations, supernatant from SPARC-primed BMDMs exposed to the same stressors markedly impaired insulin secretion from MIN6 cells (Fig. 6b). Interestingly, although SPARC did not alter the transcription of inflammatory cytokines or the secretion of TNF-α under stress conditions, it significantly enhanced the release of mature IL-1β (Fig.6c; Supplementary Fig. S3i and j).

Given that IL-1β is a canonical downstream effector of NLRP3 inflammasome activation [53] and that SPARC has been reported to enhance NLRP3-mediated pro-inflammatory responses in macrophages [38], we hypothesized that SPARC selectively activates this pathway to drive β-cell dysfunction. To test this hypothesis mechanistically, we assessed the proteolytic activation of caspase-1 and subsequent maturation of IL-1β. In LPS-primed BMDMs, SPARC pre-treatment significantly enhanced the cleavage of pro-caspase-1 into its active p20 subunit and increased the secretion levels of mature IL-1β into the culture medium (Fig. 6d). Furthermore, IF imaging demonstrated that SPARC pre-exposure markedly increased the formation of ASC specks in BMDMs upon LPS stimulation, a hallmark of inflammasome complex assembly [54], while having minimal effect under basal conditions (Fig. 6e). Finally, we integrated these findings with our *in vivo* transcriptomic data. Analysis of primary islet transcripts revealed that genes associated with the NLRP3 inflammasome pathway were significantly upregulated in islets from HFD-fed mice but were effectively normalized following exercise intervention (cutoff of *P*-value < 0.05, |log_2_(fold change)| > 0.5; Fig. 6f). Collectively, our data support the involvement of SPARC-associated NLRP3–IL-1β signaling in islet inflammation under chronic metabolic stress. Exercise intervention is associated with attenuation of this pathway.

### Circulating SPARC levels correlate with insulin resistance and β-cell dysfunction in patients with T2D

**Figure 7:**
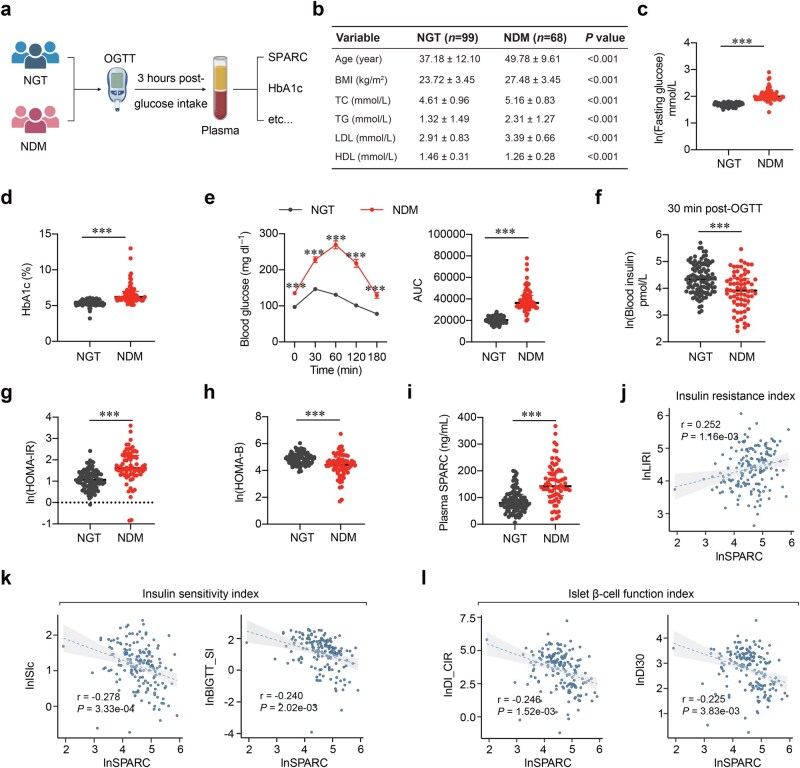
Circulating SPARC levels correlate with metabolic dysfunction in humans with T2D. (a) Schematic representation for experimental procedures for (b–l) in a population study from a community-based cohort. (b) Information of control human subjects with NGT or patients with NDM. (c) Fasting glucose measurement. Data represent mean ± SEM. ***P < 0.001; two-tailed unpaired Mann-Whitney test. (d) HbA1c percentage measurement. Data represent mean ± SEM. ***P < 0.001; two-tailed unpaired Mann–Whitney test. (e) OGTT measurement (left). Data represent mean ± SEM. ***P < 0.001; two-way ANOVA with Tukey’s multiple comparison test. AUC quantification of OGTT results (right). Data represent mean ± SEM. ***P < 0.001; two-tailed unpaired Student’s *t*-test. (f) Measurements of 30-min post-OGTT serum insulin. Data represent mean ± SEM. ***P < 0.001; two-tailed unpaired Mann–Whitney test. (g) HOMA-IR measurement. Data represent mean ± SEM. ***P < 0.001; two-tailed unpaired Mann-Whitney test. (h) HOMA-B measurement. Data represent mean ± SEM. ***P < 0.001; two-tailed unpaired Mann–Whitney test. (i) Measurements of plasma SPARC levels. Data represent mean ± SEM. ***P < 0.001; two-tailed unpaired Mann–Whitney test. (j) Correlation analysis between plasma SPARC levels and insulin resistance index. (k) Correlation analyses between plasma SPARC levels and insulin sensitivity indices. (l) Correlation analyses between plasma SPARC levels and islet β-cell function indices. For data shown in (b–l), *n* = 99 healthy individuals in NGT group; *n* = 68 T2D individuals in NDM group. Variables in (c), (f–h), and (j–l) were analyzed on a natural log scale. AUC, area under the curve; DI_CIR, corrected insulin response divided by HOMA-IR; DI30, 30-min post-OGTT blood insulin levels multiplied by Matsuda’s insulin sensitivity index composite; ISIc, Matsuda’s insulin sensitivity index composite; LIRI, liver insulin resistance index.

To evaluate the clinical relevance of our findings in human metabolic disease, we analyzed blood samples from a cohort of 167 individuals, including 99 healthy control subjects with normal glucose tolerance (NGT) and 68 patients with newly diagnosed type 2 diabetes mellitus (NDM) (Fig. 7a and b). As expected, patients with NDM exhibited pronounced metabolic dysregulation, cha­racterized by significantly elevated fasting blood glucose (Fig. 7c), higher glycated hemoglobin (HbA1c) (Fig. 7d), and impaired glucose tolerance during oral glucose tolerance test (OGTT) (Fig. 7e). Furthermore, the NDM group displayed a significant reduction in serum insulin levels at 30-min post-OGTT (Fig. 7f). In alignment with these glycemic disturbances, the NDM group showed a marked increase in the Homeostatic Model Assessment of Insulin Resistance (HOMA-IR) (Fig. 7g), and a decrease in β-cell secretory function as indicated by the Homeostatic Model Assessment of β-cell function (HOMA-B) index (Fig. 7h). Notably, circulating SPARC concentrations, quantified via ELISA, were significantly elevated in NDM patients compared to NGT controls (Fig. 7i), suggesting a systemic association between circulating SPARC and diabetic status.

To further explore the relationship between SPARC and glucose homeostasis, we performed a series of clinical correlation analyses. Plasma SPARC levels exhibited a robust positive correlation with HOMA-IR, indicating a strong link between this circulating factor and systemic insulin resistance (Fig. 7j). Conversely, elevated SPARC levels were negatively correlated with multiple indices of insulin sensitivity (Fig. 7k). More importantly, we observed a significant inverse correlation between circulating SPARC and key indices of β-cell function (Fig. 7l). These clinical data support an association between circulating SPARC and T2D-related metabolic phenotypes, while not establishing causality or therapeutic utility.

## Discussion

The global escalation of T2D underscores the urgent need of the molecular mechanisms governing metabolic resilience. While contemporary clinical management of T2D emphasizes systemic glycemic control [55, 56], our study suggests that exercise intervention during the compensatory phase of T2D conferred direct protection to pancreatic islets. Consistent with previous findings that the islets represent dynamic immunometabolic niches rather than localized endocrine clusters [2–4], we demonstrate that the therapeutic benefits of exercise were closely linked to remodeling of the islet immune microenvironment. Utilizing an integrative multi-omics pipeline, we show that exercise intervention improved β-cell in T2D likely through restoring the homeostatic interactions between intra-islet immune cells and β-cells.

Although the benefits of exercise on peripheral insulin sensitivity are well-established [57, 58], our findings delineate a direct and profound impact of physical activity on islet architecture and β-cell plasticity. Notably, our previous study showed that high-intensity treadmill training exerted a more robust effect than voluntary wheel running, suggesting a threshold-dependent mechanism for islet remodeling [20]. This exercise-mediated restoration is supported by the normalization of GSIS, as well as intracellular Ca^2+^ signaling, a hallmark of functional GSIS coupling [59, 60]. Our single-cell transcriptomic profiling further revealed that this remodeling was characterized by a favorable shift in β-cell subpopulations, specifically by limiting the expansion of the dysfunctional β2 subcluster. This subset, featured by the upregulation of stress-responsive genes such as *Dapl1* and *Pdyn* [45, 46], typically expands at the expense of the protective β1 subcluster, which is enriched in *Mt1*/*Mt2* and *Nupr1* [42–44]. By restoring this subpopulation balance, exercise preserved the protective, *Mt1*/*Mt2*/*Nupr1*-enriched β1 subcluster, thereby alleviating ER stress, reinforcing β-cell identity, and enhancing β-cell GSIS function. Importantly, this cellular plasticity was closely associated with the attenuation of chronic islet inflammation. By reducing CD45^+^ leukocyte infiltration and reversing the pro-inflammatory remodeling of the immune niche, exercise effectively re-established a homeostatic milieu that allowed β-cell to preserve their identity and secretory function. These findings suggest that restoration of the protective β1 subcluster is not an isolated event but is associated with a rejuvenated immune microenvironment in which suppression of inflammatory signaling helps improve functional GSIS coupling β-cells.

A key finding of this study is the identification of SPARC as a potential exercise-regulated factor that links peripheral metabolic stress to islet dysfunction. Beyond its established roles in extracellular matrix remodeling and adipose tissue inflammation [38, 61], our data implicate SPARC as an endocrine factor in T2D pathogenesis. Through proteomic prioritization, we identify SPARC as a candidate characterized by reciprocal regulation, induction by HFD and suppression by exercise, reflecting a systemic metabolic recalibration across the liver, skeletal muscle, and adipose tissue. Consistent with previous population studies [62], our clinical cohort study further supports the pathophysiological relevance of these findings in humans. The elevation of circulating SPARC in patients with T2D, together with its positive correlation with insulin resistance and inverse correlation with insulin sensitivity as well as β-cell function indices, provides a promising translational basis for further investigation. However, we acknowledge that our initial proteomic screen focused on a specific molecular weight window of 25–45 kDa, which may have excluded larger signaling complexes or high-molecular-weight factors (> 100 kDa). Future secretome mapping across broader mass ranges will be warranted to more fully delineate the landscape of exercise-regulated systemic mediators.

Mechanistically, our data support a contributory role for the SPARC–macrophage axis in β-cell failure under metabolic stress. We provide evidence that SPARC neither directly interacts with β-cells nor modulates their insulin secretion function, but instead targets islet-resident macrophages. Through Csf1r antibody-mediated depletion, we demonstrate that these myeloid cells are requisite sensors through which extracellular SPARC impairs insulin secretion. Notably, our work helps reconcile a potential divergence with previous literature regarding SPARC-mediated inflammation. While a prior study in adipose tissue demonstrated that SPARC promotes the NLRP3 inflammasome activation at the priming step via JNK/p38 signaling [38], our results emphasize that SPARC exerts a potent effect on the subsequent activation stage. This is evidenced by the marked enhancement of caspase-1 proteolytic cleavage and mature IL-1β secretion, even in the absence of *Il-1β* transcriptional induction. This resultant IL-1β release may perpetuate a vicious cycle of intra-islet inflammation, oxidative stress, and β-cell dysfunction, consistent with clinical observations in human T2D islets [63–65]. These findings suggest that SPARC-driven priming and activation of inflammasome may operate in sequence and in a context-dependent manner. Thus, while our work emphasized the enhanced caspase-1 activation as a prominent feature in islet macrophages, we do not rule out the involvement of the priming step in our model. It is highly possible that SPARC acts as a multi-modal regulator that reinforces both the priming and activation signals to promote inflammasome-mediated β-cell dysfunction.

Our validation in primary human islets further supports the clinical relevance of SPARC-associated macrophage–β-cell axis. Despite the inherent heterogeneity among human donors, including differences in age, sex, and metabolic history, exogenous SPARC consistently impaired insulin secretion in primary human islets. While the sample size is a recognized limitation, the robustness and consistency of this deleterious effect across different donors underscores the pathological relevance of SPARC to human β-cell health. Regarding the genetic models for studying SPARC, we acknowledge that loss-of-function genetic models are valuable tools for establishing necessity. However, whole-body Sparc deficiency may introduce confounding developmental defects and systemic imbalances. Therefore, conditional and tissue-specific knockout models will be required to rigorously define the contribution of SPARC from distinct metabolic tissues to islet dysfunction and exercise-mediated protection. We have initiated the development of such models to determine whether tissue-specific SPARC ablation can recapitulate the islet-protective effects of exercise.

In conclusion, our study delineates the remodeling of the islet immune microenvironment as an important mechanism through which exercise preserves β-cell function during T2D progression. We further show that exercise-associated suppression of circulating SPARC may contribute to reduced inflammatory signaling in macrophages and improved β-cell function. These findings provide a framework for further mechanistic and translational investigation into exercise-regulated immunometabolic pathways in T2D.

### Limitations of the study

Despite the insights provided by our findings, several limitations should be acknowledged. First, although we show that SPARC levels are elevated under metabolic stress and signifi­cantly suppressed by exercise, this study did not include *in vivo* SPARC-neutralizing experiments or genetic loss-of-function/gain-of-function models. Consequently, it remains to be definitively established whether *in vivo* SPARC ablation alone is sufficient to mimic the beneficial effects of exercise on β-cell function, and whether SPARC overexpression blunts the exercise-induced islet protection. Second, while we identified a macrophage-dependent mechanism, this is supported mainly by *ex vivo* macrophage depletion and cell-based assays. Further validation using *in vivo* myeloid-specific genetic perturbations would be required to confirm the precise role of immuno–metabolic crosstalk in this context. Third, given that SPARC is multi-organ derived, the dominant tissue source responsible for the exercise-induced decrease in circulating SPARC under metabolic stress remains unresolved in this study. Finally, our functional validation in human islets was limited by a small sample size from only two non-diabetic donors. Furthermore, since our human cohort data are cross-sectional, they support a strong clinical association between circulating SPARC and T2D pathogenesis but do not, at this stage, establish direct causality or immediate therapeutic utility. Future research employing longitudinal designs and larger-scale functional stu­dies is necessary to further validate these findings.

## Materials and methods

### Human population study

We recruited volunteers in Jurong, Jiangsu Province, China, for screening of glucose metabolism [66]. All participants underwent a 75-g OGTT with blood sampling at five time points. Venous blood samples were collected before glucose ingestion (0 min) and at 30, 60, 120, and 180 min after oral administration of 75 g anhydrous glucose for measurement of serum glucose, insulin, and C-peptide levels. Ultimately, 99 individuals with NGT and 68 patients with NDM were included in the analysis (Fig. 7b). Human population-based studies were approved by the Ethics Committee of the First Affiliated Hospital of Nanjing Medical University (2021-SR-298). All participants provided written informed consent. Based on the glucose and insulin measurements obtained during the OGTT, we calculated multiple indices for the assessment of insulin resistance, insulin sensitivity, and islet β-cell function. HOMA-IR and HOMA-β were calculated using fasting plasma glucose and fasting insulin concentrations. The liver insulin resistance index (LIRI) and insulin sensitivity indices, including the composite insulin sensitivity index (ISI_composite, ISIc) and the β-cell function, insulin sensitivity, and glucose tolerance test (BIGTT) insulin sensitivity index (BIGTT-SI), were calculated as previously reported [67, 68]. Disposition indices, including DI_CIR and DI_30, were used to evaluate pancreatic β-cell secretory function after adjustment for insulin sensitivity [69, 70].

### Animal models

WT C57BL/6J mice were procured from GemPharmatech Co., Ltd (Nanjing, China). All animal procedures were conducted in accordance with guidelines approved by the Animal Care and Use Committee of Zhejiang University. Mice were maintained under a 12-h light/12-h dark cycle at 23°C, and age-matched male mice were used throughout the study. To establish a diet-induced obesity model, 12-week-old male C57BL/6 J mice were fed either a standard chow diet (10% fat, 70% carbohydrate, and 20% protein; Jiangsu Xietong Pharmaceutical Bio-engineering, 1010088) or an HFD (60% fat, 20% carbohydrate, and 20% protein; Research Diets, D12492) for 12 weeks. For exercise intervention, mice that had been maintained on the HFD for 12 weeks underwent treadmill running training for an additional 6–10 weeks.

### GTT and ITT

GTT and ITT were conducted as previously described [71]. For GTT, mice were fasted overnight (approximately 16 h) and administered glucose (1.5 g/kg body weight) via intraperitoneal injection, with glucose concentrations adjusted to ensure consistent injection volumes across mice. Blood glucose was measured from tail snip samples at baseline (0 min) and at 15, 30, 60, and 120 min post-injection. For ITT, mice were fasted for 4 h and injected intraperitoneally with insulin (1.2 U/kg body weight), with insulin concentrations similarly adjusted to standardize injection vo­lume. Blood glucose levels were assessed at the same time points as in GTT.

### Human islets

Primary human islets were isolated from one non-diabetic male donor and one non-diabetic female donor. Islets were cultured in CMRL 1066 (X01810; icelltrans), supplemented with 10% fetal bovine serum (FBS), 100 U/mL penicillin, 100 μg/mL streptomycin, 10 U/mL heparin sodium, 10 mmol/L glutamine, and 0.1 U/mL insulin. Due to the limited availability of primary human pancreatic islets, functional experiments were performed using islets from only two non-diabetic donors (one male and one female). We acknowledge this as a limitation of the study. Detailed donor characteristics (including age, body mass index, and cold ischemia time) are available from the corresponding author upon reasonable request. Inter-donor variability in metabolic status, age, and sex may influence islet sensitivity to SPARC, and larger multi-donor cohorts will be required in future studies to fully address this. The isolation of human islets for *ex vivo* protein administration and functional analysis was performed in the Second Affiliated Hospital of Zhejiang University under the informed consent of deceased organ donors’ family (Approval number: 2022-0978).

### Isolation of primary murine islets

Mouse islets were isolated from the pancreas of mice, anesthetized through intraperitoneal injection of 2% pentobarbital sodium (P3761; Sigma-Aldrich), digested with intraductal collagenase (Collagenase type V, 1 mg/mL, C9263; Sigma-Aldrich), followed by purification with Histopaque 1119 (11191; Sigma-Aldrich) density gradient centrifugation. Purified islets were subsequently hand-selected under a stereo microscope. The isolated islets were then incubated overnight in RPMI-1640 medium (11875-093; Gibco) containing 50 μg/mL streptomycin, 50 U/mL penicillin, and 10% FBS in a humidified incubator at 37 °C with 5% CO_2_ for further analysis. In some experiments, islets were co-incubated with 60 µg/mL CSF-1R blocking antibody anti-CD115 (A2159; Selleck).

### Intracellular calcium imaging of primary islets

Primary murine islets were isolated and cultured overnight, and then washed twice with Krebs buffer containing 2 mmol/L glucose. The islets were loaded with Fluo-4 AM (5.6 µmol/L; F14201, Thermo Fisher Scientific) in Krebs buffer supplemented with 0.4‰ Pluronic F-127 (P2443; Sigma-Aldrich) and 2 mmol/L glucose for 45 min. After loading, islets were washed again with low-glucose Krebs buffer (2 mmol/L), transferred to a 35-mm glass-bottom dish (MatTek), and allowed to stabilize for 15 min. Live imaging was performed using a Zeiss LSM 800 inverted confocal microscope equipped with a humidified 37°C chamber supplied with 5% CO_2_. Image acquisition began 2 min after raising the glucose concentration to a final of 16.8 mmol/L, and time-lapse images were captured every 10 s until fluorescence intensity reached a plateau. Fluorescence dynamics were analyzed using ImageJ (version 1.52q; NIH).

### Islet perifusion assay

Primary murine islets were isolated and cultured overnight prior to perifusion assays. For each experiment, approximately 150 islet equivalents were loaded into a perifusion chamber. The islets were initially perifused for 30 min with Krebs buffer (135 mmol/L NaCl, 3.6 mmol/L KCl, 0.5 mmol/L NaH_2_PO_4_, 1.5 mmol/L CaCl_2_, 2 mmol/L NaHCO_3_, 10 mmol/L HEPES, and 0.1% BSA, pH 7.4) containing 2.8 mmol/L glucose. This was followed by sequential perifusion with Krebs buffer at 2.8 mmol/L glucose (25 min), 16.8 mmol/L glucose (30 min), 2.8 mmol/L glucose (20 min), and 30 mmol/L KCl (20 min), at a constant flow rate of 1 mL/min. Effluent fractions were collected at 1-min intervals. After the assay, islets were recovered from the chamber for total protein quantification using the BCA assay. Insulin content in each fraction was measured with an HTRF insulin assay kit (62INSPEC; Cisbio) according to the manufacturer’s protocol and normalized to total protein. To assess biphasic insulin secretion, Phase I and Phase II were deli­neated based on the plateau onset of Phase II, and the AUC was calculated for each phase.

### H&E staining of murine pancreas tissue and quantification

Freshly isolated mouse pancreatic tissues were fixed in 4% paraformaldehyde (PFA; P6148; Sigma-Aldrich) at 4°C for 16 h, followed by paraffin embedding and sectioning at a thickness of 3 μm. After H&E staining, the tissue sections were subjected to whole-slide scanning using a digital slide scanner (KFBIO PRO-400, Jiangfeng Bio). The islet area and total pancreatic area on each section were quantified with K-Viewer software (version 1.7.1.1) for ratio calculation. A total of 20–30 pancreatic sections (4–6 mice per group, with five sections collected from each mouse at intervals of at least 50 μm) were included for islet area quantification.

### IF staining

Freshly isolated mouse pancreatic tissues were fixed in 4% PFA (P6148; Sigma-Aldrich) at 4°C for 16 h, followed by paraffin embedding and sectioning at a thickness of 4 μm. Tissue sections were deparaffinized in xylene and rehydrated through a graded ethanol series (100%, 95%, and 70%), then rinsed with double-distilled water. Antigen retrieval was performed by incubating the sections in sodium citrate buffer (E673001; Sangon Biotech) at 95°C for 20 min in a water bath. After cooling to RT, the sections were washed with PBS and blocked with 10% BSA (36106ES25; Yeasen) in PBS for 1 h at room temperature. Subsequently, the sections were incubated overnight at 4°C with insulin primary antibody (1:10,000; homemade) diluted in PBS containing 5% BSA. Freshly collected mouse pancreatic tissues were embedded in OCT compound (SAKURA Tissue-Tek) and frozen at −80°C before sectioning. Tissue sections were fixed with 4% PFA for 10 min, followed by permeabilization with 0.3% Triton X-100 (A110694-0500; Sangon Biotech) for 10 min. The sections were then blocked with 10% BSA for 1 h and incubated overnight at 4°C with APC-conjugated anti-CD45 antibody (1:200; 103112; BioLegend). For fixed-cell imaging experiments, BMDMs were seeded on coverslips and treated with 0.68 μmol/L SPARC-Fc or Fc proteins for 24 h, followed by treatment with or without LPS (1 μg/mL; L4391; Sigma) for 4 h, and then stimulated with nigericin (10 μmol/L; tlrl-nig; InvivoGen) for 1.5 h. Subsequently, the cells were fixed with 4% PFA (P6148; Sigma-Aldrich) in PBS for 15 min. After fixation, cells were blocked with 1% BSA in PBS for 30 min, followed by overnight incubation at 4°C with rabbit anti-ASC antibody (1:200; AG-25B-0006; Adipogen). The following day, after three washes with PBS, the sections were incubated with Alexa Fluor-conjugated secondary antibody (1:200; Invitrogen) for 1.5 h at room temperature in the dark. Nuclei were counterstained with DAPI (1:2000; P3693; Invitrogen), and the sections were mounted with Fluoromount-G mounting medium (36307ES08; Yeasen) and sealed with nail polish. Images were acquired using ZEISS LSM 900 and quantified by ImageJ 1.52q software (NIH).

### RNA-seq analysis

RNA samples were sent to the BGI group (Wuhan, China) for library preparation and sequencing. Briefly, mRNAs were isolated from total RNA and fragmented. These fragmented mRNAs were used for reverse transcription and second-strand cDNA synthesis. Following this, adenine tailing and adaptor ligation procedures were employed to prepare the cDNAs for PCR amplification and subsequent sequencing. The sequencing itself was executed in the paired-end mode on the BGISEQ-500 system. Data were processed with the standard BGI mRNA analysis pipeline. Gene expression levels were quantified as FPKM, and statistical analysis was conducted using the DESeq2 (v.1.20.0) package. GO and pathway classification, along with enrichment analysis, were performed using clusterProfiler (V3.12.0).

### Single-cell preparation of primary islets

Mouse islets were dispersed into single cells by digestion with TrypLE™ Express Enzyme (12604013; Gibco) at 37°C for 15 min, followed by the addition of an equal volume of PBS (2 mmol/L EDTA, 10% FBS) to terminate digestion, and then rinsed twice with PBS.

### Flow cytometry analysis

The staining buffer was PBS containing 0.5% BSA and 2.5 mmol/L EDTA. Immunostaining was performed with the primary antibody CD45 (110729; BioLegend), along with Fixable Viability Dye eFluor™ 450 (65–0863-14; Thermo Fisher) for dead cell exclusion. Stained cells were analyzed by a three-laser flow cytometer (Agilent Novocyte). Data were analyzed with Flowjo v10 software.

### scRNA-seq analysis

Isolated single-cell suspensions were subjected to droplet-based massively parallel scRNA-seq using the Chromium Single Cell 5’ Reagent Kit according to the manufacturer’s instructions. The reference genome was constructed using Celescope rna mkref and the mouse genome reference sequence version mm10. Then, the upstream analysis of single-cell transcriptome other than mkref was automatically completed through multi_rna, and the quantification results of expression levels were obtained. Subsequently, due to additional sequencing of the same batch of cells with insufficient depth, the cells with the same unique molecular identifier were first merged, and the counts were summed. Cells with less than 1000 and more than 4000 detected genes were excluded, and cells with mitochondrial gene expression levels accounting for more than 5% were filtered. The Seurat (V4.3.0) package was used for subsequent dimensionality reduction and clustering. After performing principal component analysis and preliminary clustering on the top 400 highly variable genes, the average expression levels of *Ins1*, *Ins2*, *Gcg*, and *Sst* in the immune cell clusters were regarded as background signals, and the background signals were removed from all cells. Then, the above process was repeated again using the top 400 highly variable genes. In particular, the first 11 principal components (PCs) were selected and the clustering resolution was set to 0.3. β-cells were further extracted, and the top 10 PCs were selected with a clustering resolution of 0.1. The detection of reversible genes of β-cells under different groups was based on the Wilcoxon test. Seurat and ggplot2 (V3.5.2) were used to plot the results. For the comparison of functional genes across different groups, the data did not meet the assumption of normality according to the Shapiro–Wilk test, therefore, the Kruskal–Wallis test and Dunn test were directly applied.

### RNA extraction and RT-qPCR analysis

Total RNA was isolated from cultured cells using TRIzol reagent (Life Technologies). Subsequently, 1 μg of RNA from each sample was reverse-transcribed into cDNA with HiScript II Q RT SuperMix for quantitative PCR (qPCR) (R222-01; Vazyme). qPCR was carried out using SYBR Green reagent (Q711-02; Vazyme) to evaluate gene expression levels. The relative expression of each target gene was calculated and normalized to internal control genes. All primer sequences used in this study are provided in Supplementary Table S1.

### Immunoblot analysis

Protein samples were prepared for Western blot analysis by lysing cultured cells with RIPA lysis buffer (P0013B; Beyotime Biotechnology). The resulting protein supernatants, together with purified protein samples, were then denatured, separated by SDS-PAGE, and transferred onto a polyvinylidene fluoride membrane (IPVH00010, ISEQ00010; Millipore), followed by immunoblotting with the following primary antibodies: mouse anti-Fc (dilution 1:1000; A-10648; Invitrogen), rabbit anti- SPARC (dilution 1:1000; 15274-1-AP; Proteintech); mouse anti-Caspase-1 (p20) (dilution 1:1000; AG-20B-0042; AdipoGen); mouse anti-IL-1β (dilution 1:1000; AF- 401-NA; R&D Systems); and rabbit anti-HSP90 (dilution 1:1000; C45G5; Cell Signaling Technology). Secondary antibodies from Sigma-Aldrich were applied as follows: anti- rabbit (dilution 1:10,000; A6154), and anti-mouse (dilution 1:10,000; A4416).

### Preparation of SPARC-Fc protein

The coding sequence of SPARC was synthesized by Synbio Technologies with an Fc tag fused to its C-terminus, and subsequently cloned into the pcDNA3.0 vector for ­subsequent experiments. Expi293F cells were transfected with the plasmid pcDNA3.0-SPARC-Fc following standard transfection protocols. At 24 h post-transfection, sodium butyrate (1 mol/L) was added at a 1:1000 dilution to inhibit cell proliferation. The cells were then cultured for an additional 4–5 days. The culture supernatant was harvested and sequentially centrifuged at 450 *g* for 5 min and 12,000 *g* for 5 min to remove residual cells and debris, respectively. SPARC-Fc protein was affinity-purified using protein A/G agarose resin (36403ES08; YEASEN), concentrated with a 30-kDa ultrafiltration device (UFC905024; Millipore), and stored at −80°C until further use.

### GSIS assay of MIN6 cells

GSIS in MIN6 cells was assessed as follows. After a 30-min preincubation in glucose-free Krebs buffer, the cells were exposed to Krebs buffer containing either 2.8 mmol/L or 16.8 mmol/L glucose for 1 h. Supernatants were then collected for insulin quantification. Cells were lysed in cold lysis buffer, and total protein content was determined by BCA assay. Insulin secretion under each condition was measured using a HTRF insulin assay kit (62INSPEC; Cisbio) according to the manufacturer’s protocol and normalized to the total protein content.

### SEAP-SPARC binding assay

The SEAP-binding assay was performed following the established protocol [72]. In brief, HEK293T cells were transiently transfected with expression vectors for SEAP or SEAP-SPARC fusion proteins. Twelve hours post-transfection, the culture medium was replaced with serum-free medium. After an additional 48 h of incubation, the supernatant was collected and concentrated using Centricon centrifugal filters (Millipore). The concentrated conditioned medium (CM) containing SEAP or SEAP-SPARC was applied to pre-prepared cell slides and incubated at room temperature for 45 min. Following incubation, the slides were washed four times with PBS containing 0.1% Tween-20 and fixed for 10 min in fixative solution (3% formaldehyde, 20 mmol/L HEPES [pH 7.4], and 60% acetone). To inactivate endogenous alkaline phosphatase, the slides were heated at 65°C for 2 h in a water bath. The enzymatic activity of the SEAP-SPARC fusion protein was then detected using NBT/BCIP substrate (Roche, 11681451001). Images were acquired and data were collected with CellSens Standard software (Olympus).

### BMDM isolation and treatment

BMDMs were isolated following an established protocol [73]. On day 6 of differentiation, cells were pretreated with 0.68 μmol/L SPARC-Fc or Fc proteins for 24 h. On day 7, cells were stimulated with either 1 μg/mL LPS or a PT mixture (consisting of 0.125 mmol/L PA [P5585; Sigma] and 12.5 ng/mL TNF-α [300-01A; PeproTech]) for 4 h, followed by treatment with 1.1 mg/mL ATP (A7699; Sigma) for 30 min. Cells or culture supernatants were then harvested for subsequent experiments.

### Co-culture and supernatant experiments

For co-culture experiments, on day 5 of BMDM differentiation, cells were collected and co-cultured with MIN6 cells at a 1:3 ratio (BMDMs to MIN6) to mimic the cellular composition of pancreatic islets. The co-cultures were then treated as described in the BMDM isolation and treatment section. For supernatant experi­ments, the supernatant was diluted 2-fold with complete MIN6 culture medium under basal conditions. For supernatant derived from LPS- or PT-treated BMDMs, a 3-fold dilution was applied. The functional response of MIN6 cells under the respective treatment conditions was ultimately assessed by GSIS assay.

### Plasma proteomics

Plasma samples were collected from three experimental groups of mice following 10 weeks of exercise intervention. For each sample, 9 mg protein was subjected to low-abundance protein enrichment using the ProteoMiner Protein Enrichment Kit (1633006; Bio-red) according to the manufacturer’s protocol. The enriched protein fractions were then separated and visualized using a silver staining kit (24612; Thermo Scientific). Protein bands exhibiting significant differential expression were excised for subsequent identification by mass spectrometry analysis.

### Statistical analysis

Statistical analyses were performed using GraphPad Prism 8. To evaluate statistical differences, two-tailed and multiple unpaired Student’s *t*-test was used for comparisons between two groups and multi-groups, while ANOVA and suitable *post hoc* analyses were employed for comparisons involving more than two groups. A *P* value of less than 0.05 was considered statistically significant. Statistical parameters and corresponding *P* values for data shown in each panel were included in the figure legends.

## Supplementary Material

loag014_Supplementary_Data

## Data Availability

RNA-seq datasets are available for the data presented in Figs. 2e, 2f, 3a, and 3c and Supplementary Fig. S2a–d. scRNA-seq datasets are available for the data presented in Fig. 2h–m and Supplementary Fig. S1d and e. Proteomic datasets are available for the data presented in Fig. 4c and Supplementary Fig. S3a. These datasets and all the original code for automatic image analysis have been deposited in the Mendeley data and are publicly accessible. All other data and image files are available from corresponding author upon reasonable request. All packages and codes used in this study are open-source and publicly available.
