# Supplementary material for: Exercise preserves β-cell function in type 2 diabetes by reshaping intra-islet macrophage–β-cell crosstalk
Source: Life Metab. 2026 May 26;5(4):loag014. doi: 10.1093/lifemeta/loag014 (PMC13313163; doi:10.1093/lifemeta/loag014)
Supplement: loag014_Supplementary_Data [file loag014_supplementary_data.zip › Supplementary_Information - tu.docx]

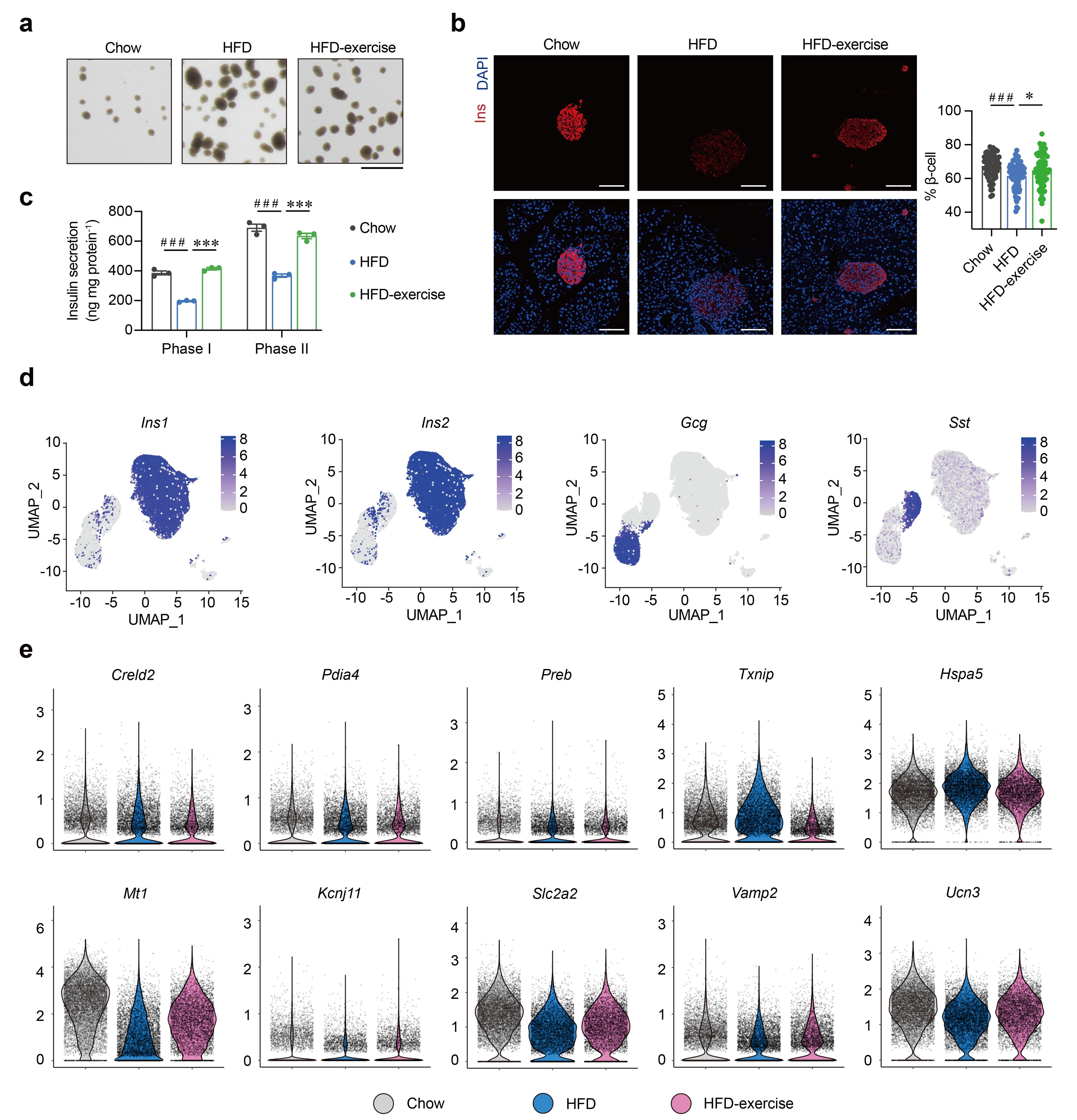


**Supplementary Figure S1** Exercise remodels islet cell composition and β-cell subclusters. (a) Representative islet images of Chow, HFD, and HFD-exercise mice. Scale bar, 1 mm. (b) Representative immunofluorescence images of mouse islets from Chow, HFD, and HFD-exercise groups (left). Scale bar, 100 µm. Quantification of β-cell percentage normalized to total islet cells from Chow, HFD, and HFD-exercise mouse islets (right). Each sample contains 5−6 mice. Data represent mean *±* SEM (dots represent 61−84 islets from each group). ^###^*P* < 0.001, HFD vs. Chow; ^*^*P* < 0.05, HFD- exercise vs. HFD; one-way ANOVA with Tukey’s multiple comparison test. (c) Biphasic insulin release levels of dynamic glucose-stimulated insulin secretion in islets from Chow, HFD, and HFD-exercise mice. Each islet sample was pooled from at least 3 animals. Data represent mean *±* SD (*n* = 3 technical replicates). ^###^*P* < 0.001, HFD vs. Chow; ^***^*P* < 0.001, HFD-exercise vs. HFD; two-way ANOVA with Tukey’s multiple comparison test. (d) scRNA-seq and UMAP visualization of islet single cells from Chow*,* HFD, and HFD-exercise groups. Expression levels of indicated marker genes are shown. Scale bar indicates gene expression level. (e) Violin plot of selected gene expression from Chow, HFD, and HFD-exercise groups. Expression level in each cell is marked in gray dot. Data in (a−c) are representative of at least two independent experiments.


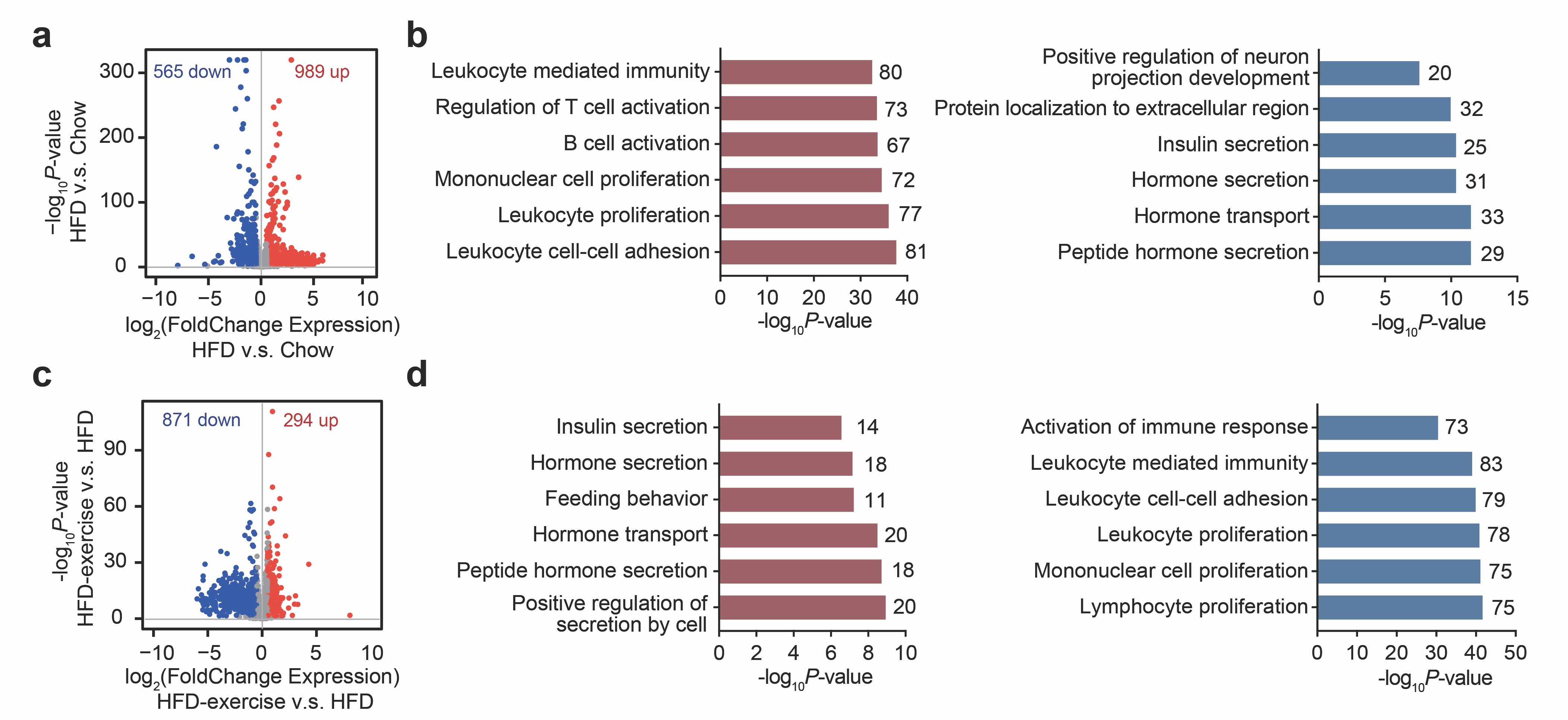


**Supplementary Figure S2** Islet RNA-seq reveals biological pathway changes following exercise intervention. (a) Volcano plot showing the gene expression difference in islets between Chow and HFD groups. Genes that are significantly upregulated (Log2FC > 0.5, *P* < 0.05, *P* values by Wald test) or downregulated (Log2FC

< −0.5, *P* < 0.05, *P* values by Wald test) were marked in red and blue, respectively. (b) GO analysis of significantly upregulated (left) and downregulated (right) genes by HFD compared to Chow group. (c) Volcano plot showing the gene expression difference in islets between HFD and HFD-exercise groups. Genes that are significantly upregulated (Log2FC > 0.5, *P* < 0.05, *P* values by Wald test) or downregulated (Log2FC < −0.5, *P* < 0.05, *P* values by Wald test) were marked in red and blue, respectively. (d) GO analysis of significantly upregulated (left) and downregulated (right) genes by HFD-exercise compared to HFD group. All panels report data in at least two independent experiments.


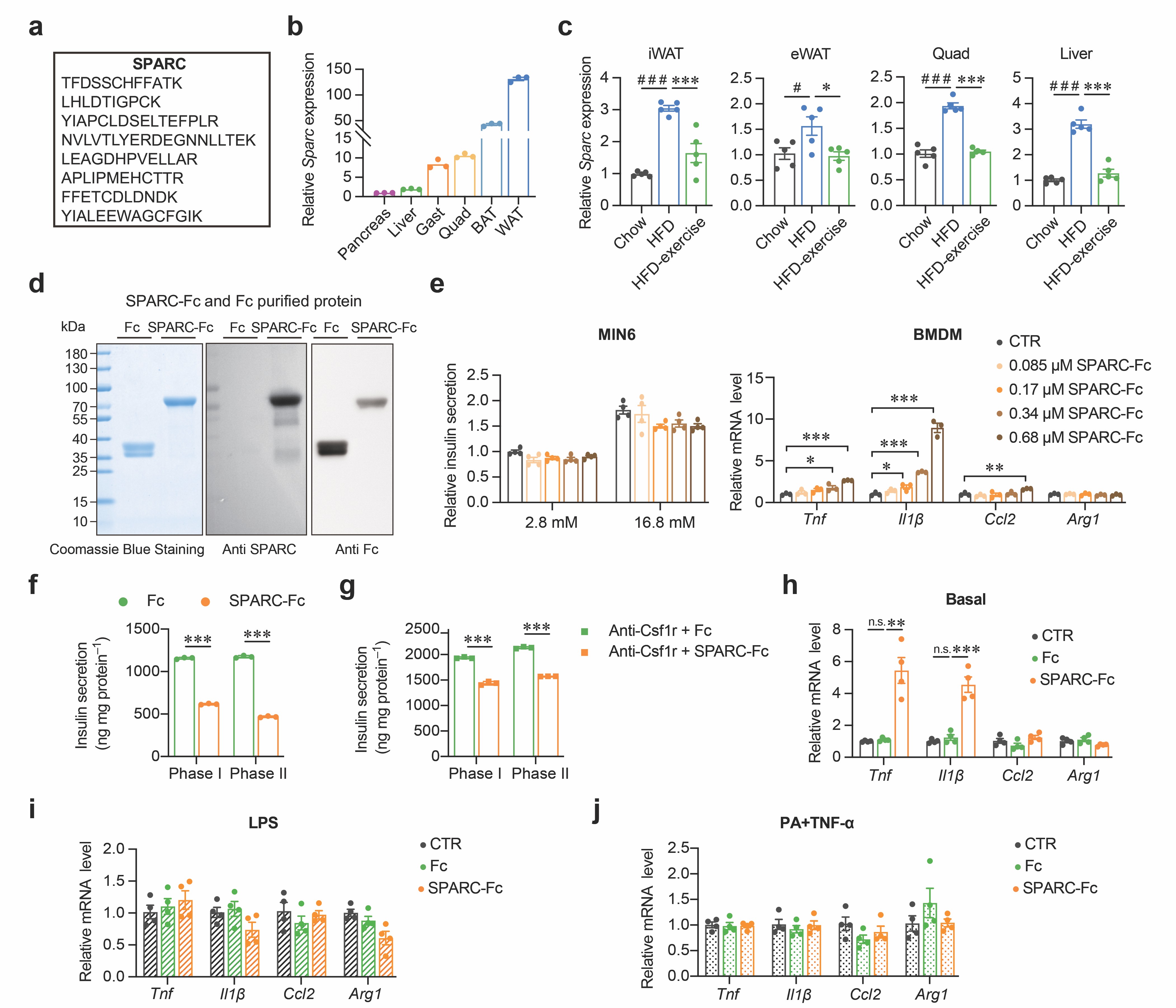


**Supplementary Figure S3** HFD-induced SPARC links islet macrophages to β-cell dysfunction. (a) The amino acid sequence of SPARC identified by mass spectrometry. (b) Tissue-wide distribution of *Sparc* mRNA in different metabolically active tissues (*n* = 3 technical triplicates). (c) Relative *Sparc* mRNA levels in iWAT, eWAT, Quad, and liver from Chow, HFD, and HFD-exercise mice. Data represent mean *±* SEM (*n* = 5 biological replicates per group)*.* ^###^*P* < 0.001; ^#^*P* < 0.05, HFD vs. Chow; ^***^*P* < 0.001; ^*^*P* < 0.05, HFD-exercise vs. HFD; one-way ANOVA with Tukey’s multiple comparison test. (d) Coomassie Brilliant Blue staining and immunoblots of purified Fc protein and Fc-tagged SPARC protein synthesized by 293F cells. (e) GSIS of MIN6 cells cultured with different concentration of SPARC-Fc protein (left). Data represent mean *±* SEM (*n* = 4 biological replicates per group); one-way ANOVA with Tukey’s multiple comparison test. Relative mRNA levels of inflammation-associated genes in BMDMs cultured with different concentrations of SPARC-Fc protein (right). Data represent mean *±* SEM (*n* = 3 biological replicates per group); ^***^*P* < 0.001; ^**^*P* < 0.01; ^*^*P* < 0.05; one-way ANOVA with Tukey’s multiple comparison test. (f) Biphasic insulin release levels of perifusion analyses on dynamic glucose-stimulated phase I and phase II insulin secretion of islets treated with SPARC-Fc or Fc proteins. Data represent mean *±* SD (*n* = 3 technical replicates per group); ^***^*P* < 0.001; two- way ANOVA with Tukey’s multiple comparison test. (g) Biphasic insulin release levels of perifusion analyses on dynamic glucose-stimulated phase I and phase II insulin secretion of anti-Csf1r antibody-mediated macrophage depleted islets treated with SPARC-Fc or Fc proteins*.* Data represent mean *±* SD (*n* = 3 technical replicates per group); ^***^*P* < 0.001; two-way ANOVA with Tukey’s multiple comparison test. (h) Relative mRNA levels of inflammation-associated genes in BMDMs cultured with SPARC-Fc protein in basal condition. Data represent mean *±* SEM (*n* = 4 biological replicates per group)*.* ^***^*P* < 0.001; ^**^*P* < 0.01; n.s., no significance; one-way ANOVA with Tukey’s multiple comparison test. (i) Relative mRNA levels of inflammation-associated genes in BMDMs cultured with SPARC-Fc protein in LPS-primed condition. Data represent mean *±* SEM (*n* = 4 biological replicates per group); one-way ANOVA with Tukey’s multiple comparison test. (j) Relative mRNA levels of inflammation-associated genes in BMDMs cultured with SPARC-Fc protein in PT-primed condition. Data represent mean *±* SEM (*n* = 4 biological replicates per group); one-way ANOVA with Tukey’s multiple comparison test. Data in (b−j) are representative of at least two independent experiments. BAT, brown adipose tissue. eWAT, epidydimal white adipose tissue. Gast, gastrocnemius muscle. iWAT, inguinal white adipose tissue. Quad, quadriceps. WAT, white adipose tissue.

**SUPPLEMENTARY TABLE**

| Gene primers | 5’ to 3’ |
| --- | --- |
| m*Rplp0* forward | GAAACTGCTGCCTCACATCCG |
| m*Rplp0* reverse | GCTGGCACAGTGACCTCACACG |
| m*Sparc* forward | GATCAGCACCCTATTGATGGG |
| m*Sparc* reverse | TCGTTGTCTAGGTCACAGGTC |
| m*Ins1* forward | AGACCATCAGCAAGCAGGTCA |
| m*Ins1* reverse | AAGTGCACCAACAGGGCC |
| m*Nkx6.1* forward | CTTCTGGCCCGGAGTGATG |
| m*Nkx6.1* reverse | GGGTCTGGTGTGTTTTCTCTTC |
| m*Abcc8* forward | GAGGGAGAAGACCCCAGGTA |
| m*Abcc8* reverse | GTGATGTTCTCCTCCACCGT |
| m*Syt7* forward | CCTGCTGGTCTCTGCAATCAT |
| m*Syt7* reverse | CAGGGTGGAGTCCTCGAAAT |
| m*Slc2a2* forward | TCATCATTGCTGGACGAAGTG |
| m*Slc2a2* reverse | TTGCCCAGAATAAAGCTGAGG |
| m*Tnf* forward | AGCCCCCAGTCTGTATCCTT |
| m*Tnf* reverse | CTCCCTTTGCAGAACTCAGG |
| m*Il1β* forward | TGGCAACTGTTCCTGAACTCAA |
| m*Il1β* reverse | AGCAGCCCTTCATCTTTTGG |
| m*Ccl2* forward | AGGTCCCTGTCATGCTTCTG |
| m*Ccl2* reverse | TCTGGACCCATTCCTTCTTG |
| m*Ccl5* forward | TGCCCACGTCAAGGAGTATTT |
| m*Ccl5* reverse | TTCTCTGGGTTGGCACACACT |
| m*NOS2* forward | GAGGCCCAGGAGGAGAGAGATCCG |
| m*NOS2* reverse | TCCATGCAGACAACCTTGGTGTTG |
| m*Arg1* forward | ACACGGCAGTGGCTTTAACC |
| m*Arg1* reverse | TGGCGCATTCACAGTCACTT |
| m*F4/80* forward | CTTTGGCTATGGGCTTCCAGTC |
| m*F4/80* reverse | GCAAGGAGGACAGAGTTTATCGTG |
| m*Mt1* forward | CCGTGGGCTGCTCCAAAT |
| m*Mt1* reverse | TAGGAAGACGCTGGGTTG |
| m*Mt2* forward | AGGAGCAGCAGCTTTTCTTG |
| m*Mt2* reverse | GACCCCAACTGCTCCTGTG |
| m*Nupr1* forward | GGACCTAGGCCTGCTTGATTC |
| m*Nupr1* reverse | CATCTTGCCCTTTTGCTGGG |
| m*Dapl1* forward | GCCATCACAAATGTCGCCAA |
| m*Dapl1* reverse | GCTTTTGATGTGCCGTGTGA |
| m*Pdyn* forward | CAGAACTGCCATAGGGGGATT |
| m*Pdyn* reverse | TAGGGTGGCCGATCCAAGAT |

**Supplementary Table S1** Primer sequences for RT-qPCR used in this study.
