# Supplementary figures and images for: Exercise preserves β-cell function in type 2 diabetes by reshaping intra-islet macrophage–β-cell crosstalk
Source: Life Metab. 2026 May 26;5(4):loag014. doi: 10.1093/lifemeta/loag014 (PMC13313163; doi:10.1093/lifemeta/loag014)

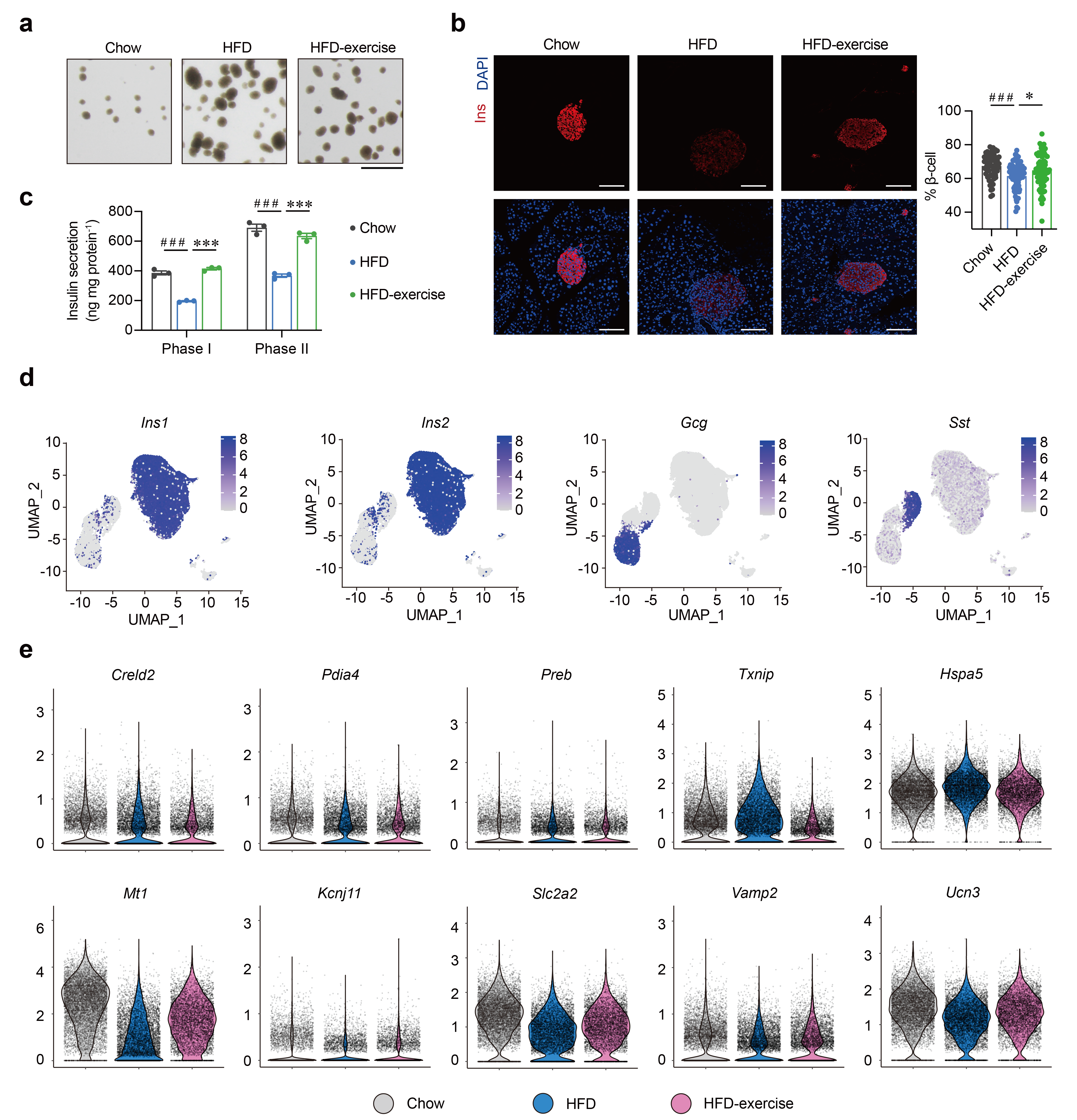

Supplement: loag014_Supplementary_Data [file loag014_supplementary_data.zip › Supplementary Figure 1.tif]

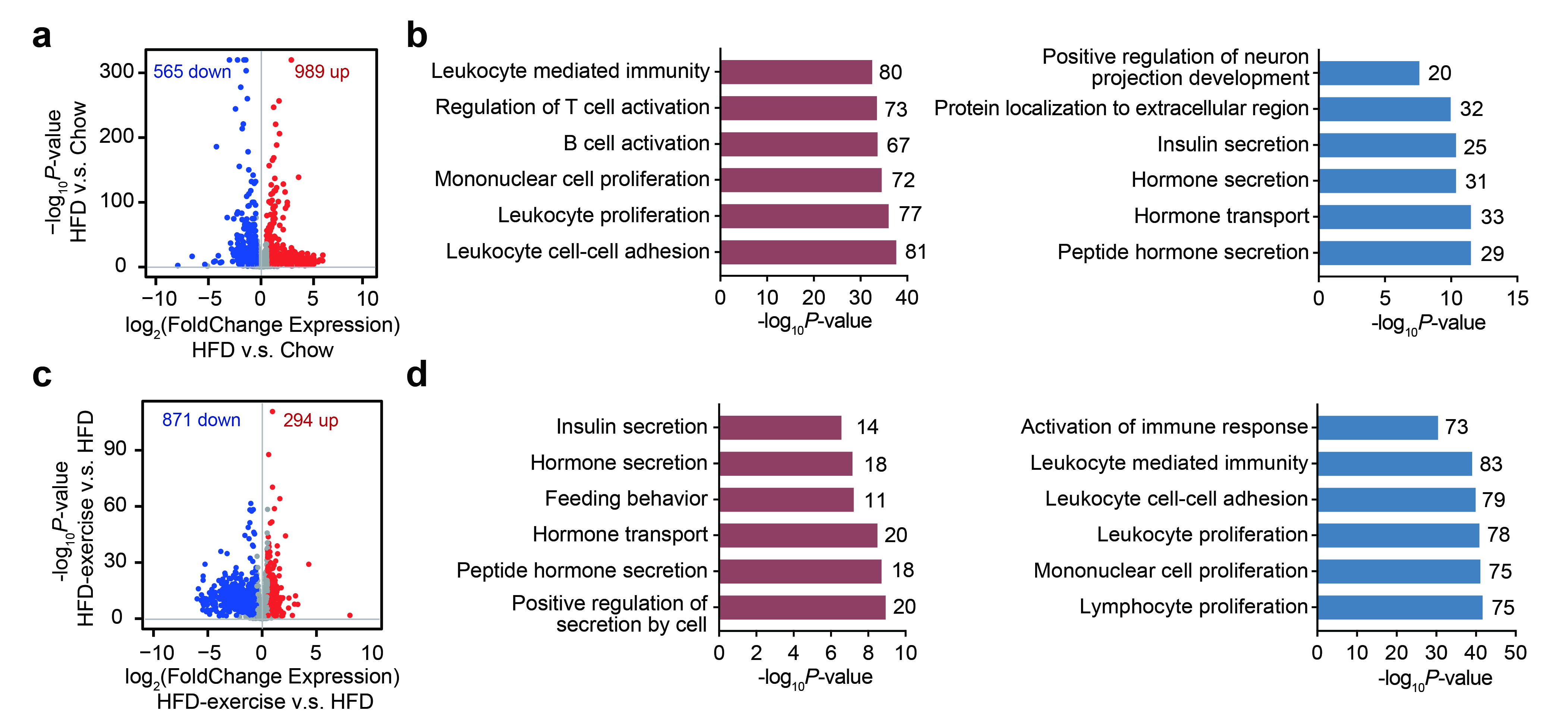

Supplement: loag014_Supplementary_Data [file loag014_supplementary_data.zip › Supplementary Figure 2.tif]

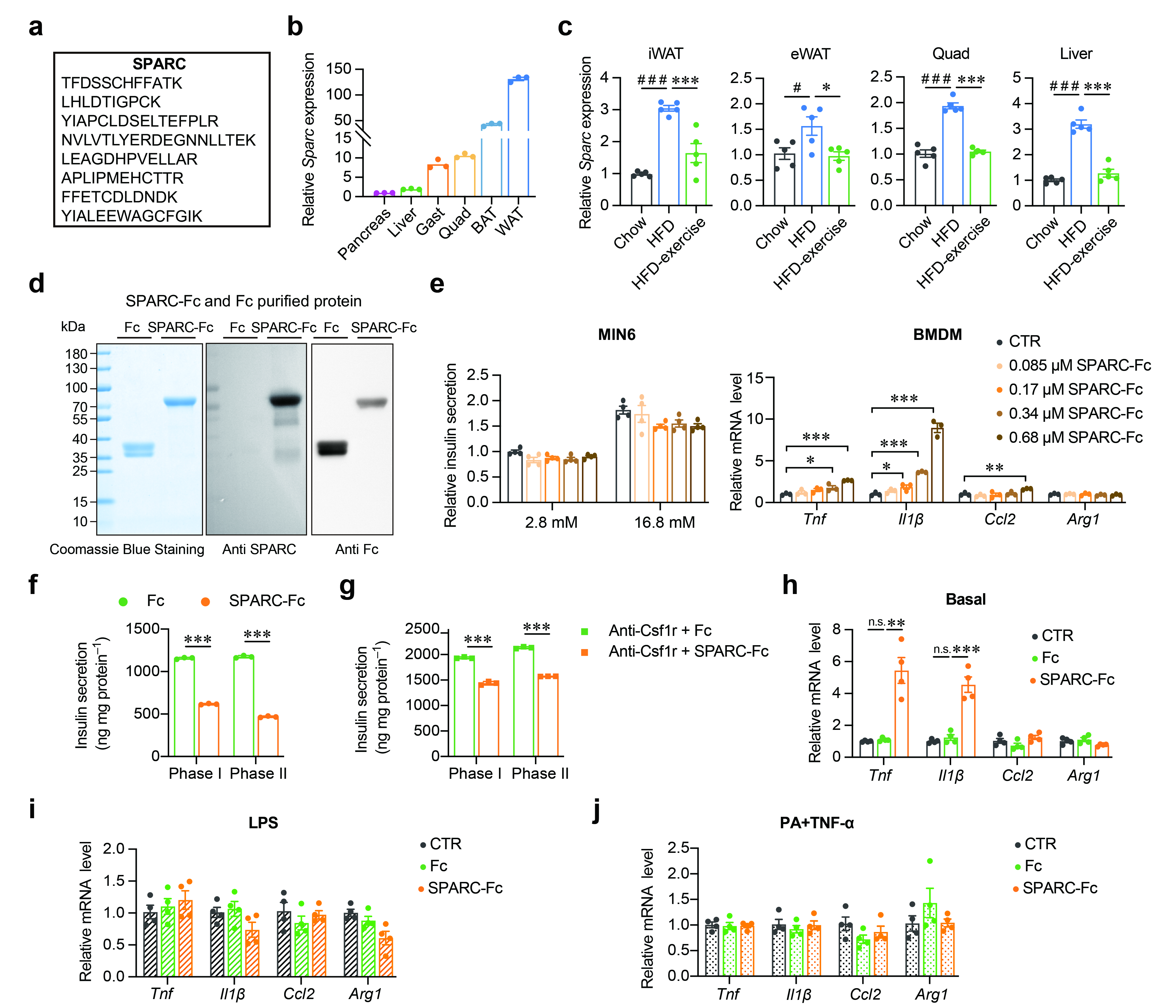

Supplement: loag014_Supplementary_Data [file loag014_supplementary_data.zip › Supplementary Figure 3.tif]
